# Health state utility values in major depressive disorder treated with pharmacological interventions: a systematic literature review

**DOI:** 10.1186/s12955-021-01723-x

**Published:** 2021-03-18

**Authors:** James Brockbank, Taryn Krause, Emily Moss, Anne Milthers Pedersen, Michael Frank Mørup, Outi Ahdesmäki, Jake Vaughan, Thor-Henrik Brodtkorb

**Affiliations:** 1 RTI Health Solutions, Manchester, UK; 2 Krause Limited, London, UK; 3 Lundbeck A/S, Valby, Denmark; 4 RTI Health Solutions, Ljungskile, Sweden

**Keywords:** Major depressive disorder, Health state utility values, Disutilities, Systematic review

## Abstract

**Background:**

Major depressive disorder (MDD) is associated with decreased patient well-being and symptoms that can cause substantial impairments in patient functioning and even lead to suicide. Worldwide, MDD currently causes the second-most years lived with disability and is predicted to become the leading cause of disability by 2030. Utility values, capturing patient quality of life, are required in economic evaluations for new treatments undergoing reimbursement submissions. We aimed to identify health state utility values (HSUVs) and disutilities in MDD for use in future economic evaluations of pharmacological treatments.

**Methods:**

Embase, PubMed, Econlit, and Cochrane databases, plus gray literature, were searched from January 1998 to December 21, 2018, with no language or geographical restrictions, for relevant studies that reported HSUVs and disutilities for patients with MDD receiving pharmacological interventions.

**Results:**

443 studies were identified; 79 met the inclusion criteria. We focused on a subgroup of 28 articles that reported primary utility data from 16 unique studies of MDD treated with pharmacological interventions. HSUVs were elicited using EQ-5D (13/16, 81%; EQ-5D-3L: 11/16, 69%; EQ-5D-3L or EQ-5D-5L not specified: 2/16), EQ-VAS (5/16, 31%), and standard gamble (1/16, 6%). Most studies reported baseline HSUVs defined by study entry criteria. HSUVs for a first or recurrent major depressive episode (MDE) ranged from 0.33 to 0.544 and expanded from 0.2 to 0.61 for patients with and without painful physical symptoms, respectively. HSUVs for an MDE with inadequate treatment response ranged from 0.337 to 0.449. Three studies reported HSUVs defined by MADRS or HAMD-17 clinical thresholds. There was a large amount of heterogeneity in patient characteristics between the studies. One study reported disutility estimates associated with treatment side effects.

**Conclusions:**

Published HSUVs in MDD, elicited using methods accepted by health technology assessment bodies, are available for future economic evaluations. However, the evidence base is limited, and it is important to select appropriate HSUVs for the intervention being evaluated and that align with clinical health state definitions used within an economic model. Future studies are recommended to elicit HSUVs for new treatments and their side effects and add to the existing evidence where data are lacking.

**Supplementary Information:**

The online version contains supplementary material available at 10.1186/s12955-021-01723-x.

## Introduction

Patients with depressive disorders can experience sadness, loss of interest or pleasure, feelings of guilt or low self-worth, disturbed sleep or appetite, feelings of tiredness, and poor concentration. These symptoms can cause substantial impairments in a patient’s ability to function and, in some cases, may lead to suicide [1]. There are two main subcategories of depressive disorders: major depressive disorder (MDD), in which patients experience major depressive episodes (MDEs), and dysthymia, which is a chronic and milder form of depression [1]. An analysis of the Global Burden of Disease database by Liu et al. [2] found that 93.7% of patients with depression in 2017 had MDD. It is estimated that MDD causes the second-most years lived with disability, after lower back pain [3]. The worldwide incidence of MDD increased from an estimated 162 million cases in 1990 to 241 million cases in 2017 [2], and MDD is predicted to become the leading cause of disability by 2030 [4].

Patients with MDD who experience an MDE can be classified based on clinical thresholds of disease severity [for example, mild, moderate, or severe as adopted in the *Diagnostic and Statistical Manual of Mental Disorders, Fifth Edition* (DSM-V)], assessed using scales such as the Hamilton Depression Rating Scale (HAMD) or the Montgomery-Åsberg Depression Rating Scale (MADRS), and on the duration of the disorder (for example, first or recurrent MDE). Major depressive disorder can be managed pharmacologically with different classes of antidepressant treatments (ADTs), including selective serotonin reuptake inhibitors (SSRIs), serotonin-norepinephrine reuptake inhibitors (SNRIs), bupropion, tricyclic antidepressants (TCAs), and monoamine oxidase inhibitors (MAOIs), as well as antipsychotics [5]. Patients who do not respond to or tolerate an initial treatment, or who relapse, usually switch to a different class of ADT or augment agents.

New pharmacological treatments are being developed to improve clinical outcomes for patients with MDD, and economic evaluations may need to be performed to assess their value. Economic evaluations are performed to assess the cost-effectiveness of the new treatments in relation to treatments already available in local health care markets. Many health care payers require cost-utility analyses that use quality-adjusted life-years (QALYs) as the main measurement of effectiveness [6]. The QALY is a generic measure of disease burden that allows comparative analyses of the value of medical interventions to be conducted. They capture both the quantity and quality of life and are calculated by multiplying time spent in certain health states with corresponding health state utility values (HSUVs) [7]; HSUVs quantify health-related quality of life (HRQoL) as a single value on a scale from 0 (dead) to 1 (perfect health). Some HRQoL instruments allow for negative values for health states worse than death. Health state utility values represent the strength of an individual’s preferences for specific health-related outcomes and can be elicited using different instruments and techniques. Discrete condition-specific health states can be measured directly using choice-based methods such as standard gamble (SG), time trade-off, and discrete-choice experiments; ranking exercises; or a visual analogue scale (VAS) [8]. Indirect measurement of HSUVs is most commonly performed using generic multi-attribute utility instruments such as the EQ-5D, Short Form six dimensions (SF-6D), and Health Utilities Index that define health states according to scores on multiple distinct domains of health. Scores are converted to HSUVs by using utility tariffs derived from general population surveys that account for public preferences. Disease-specific instruments can be used to measure HSUVs in a similar way or by mapping results to a generic instrument. Many European health technology assessment (HTA) bodies prefer that HSUVs be measured indirectly by using generic, preference-based instruments, with the EQ-5D being the most popular instrument [9]. Across 25 European countries with pharmacoeconomic guidelines, only two countries prefer HSUVs to be measured directly [6].

Economic models used to assess the cost-effectiveness of new treatments need to capture health states experienced by patients with MDD throughout the course of the disease. Health states used in cost-utility models can include different severities of depression (i.e., mild, moderate, and severe), different levels of treatment response (i.e., remission, response, and no response or refractory), a return to normal health (i.e., recovery), and disease progression (i.e., relapse and recurrence). Health states can be defined by thresholds in clinical scores such as HAMD and MADRS. Transition probabilities calculated using efficacy data from clinical trials and published, long-term outcome data are used to predict the movement of patients between the modelled health states over time. Time spent in each health state is multiplied with the corresponding HSUV to calculate QALYs of patients receiving each treatment being assessed.

The aim of this systematic literature review was to identify published HSUVs and disutility values for treatment-related adverse events that can be used to populate future economic models of pharmacological treatments for adult patients with MDD. Furthermore, we set out to highlight gaps in the evidence base and discuss considerations for cost-utility analyses informing reimbursement decisions.

## Methods

A systematic literature review was performed, using a prespecified protocol, to identify utility studies for adult patients with MDD receiving pharmacological treatment, including those on adjunctive treatment. Literature searches were conducted in PubMed, Embase, Econlit, and Cochrane databases from January 1, 1998, to December 21, 2018. Literature search strategies were designed using Medical Subject Headings (MeSH) and free-text terms (see Additional files 1–4). References of identified systematic literature reviews and cost-effectiveness analyses were searched to identify primary utility studies. Additionally, the websites of health technology agencies and relevant conferences were searched. These included the National Institute for Health and Care Excellence (NICE); the Scottish Medical Consortium; the Canadian Agency for Drugs and Technologies in Health; the National Institute for Health Research, Health Technology Assessment Database; the International Society for Pharmacoeconomics and Outcomes Research (ISPOR); the Tufts Cost-Effectiveness Analysis Registry; and the American Psychiatric Association. Conference abstracts from ISPOR meetings were indexed in Embase at the time of the searches, so separate hand searches were not performed for this conference.

For inclusion, studies were required to be conducted in adults (aged ≥ 18 years) with MDD receiving pharmacological treatment, published in English, and to report utility or disutility estimates. Excluded from the review were studies that included children (aged < 18 years), studies in which patients received only non-pharmacological interventions, studies that reported quality-of-life data only, and conference abstracts published before 2016.

Screening was performed by one researcher, with a random 20% quality check performed by a second researcher, using the predefined inclusion and exclusion criteria (see Additional file 5). Screening was conducted in two stages; at level 1, titles and abstracts were screened for eligibility, and at level 2, full-text articles of those included at level 1 were obtained and screened to confirm eligibility. If an agreement could not be reached on the eligibility of a study, a third researcher was consulted to reach consensus on the eligibility of the study. One researcher extracted data from the eligible studies included in the review. A second researcher performed a quality check of all extracted data back to the original source.

## Results

**Fig. 1:**
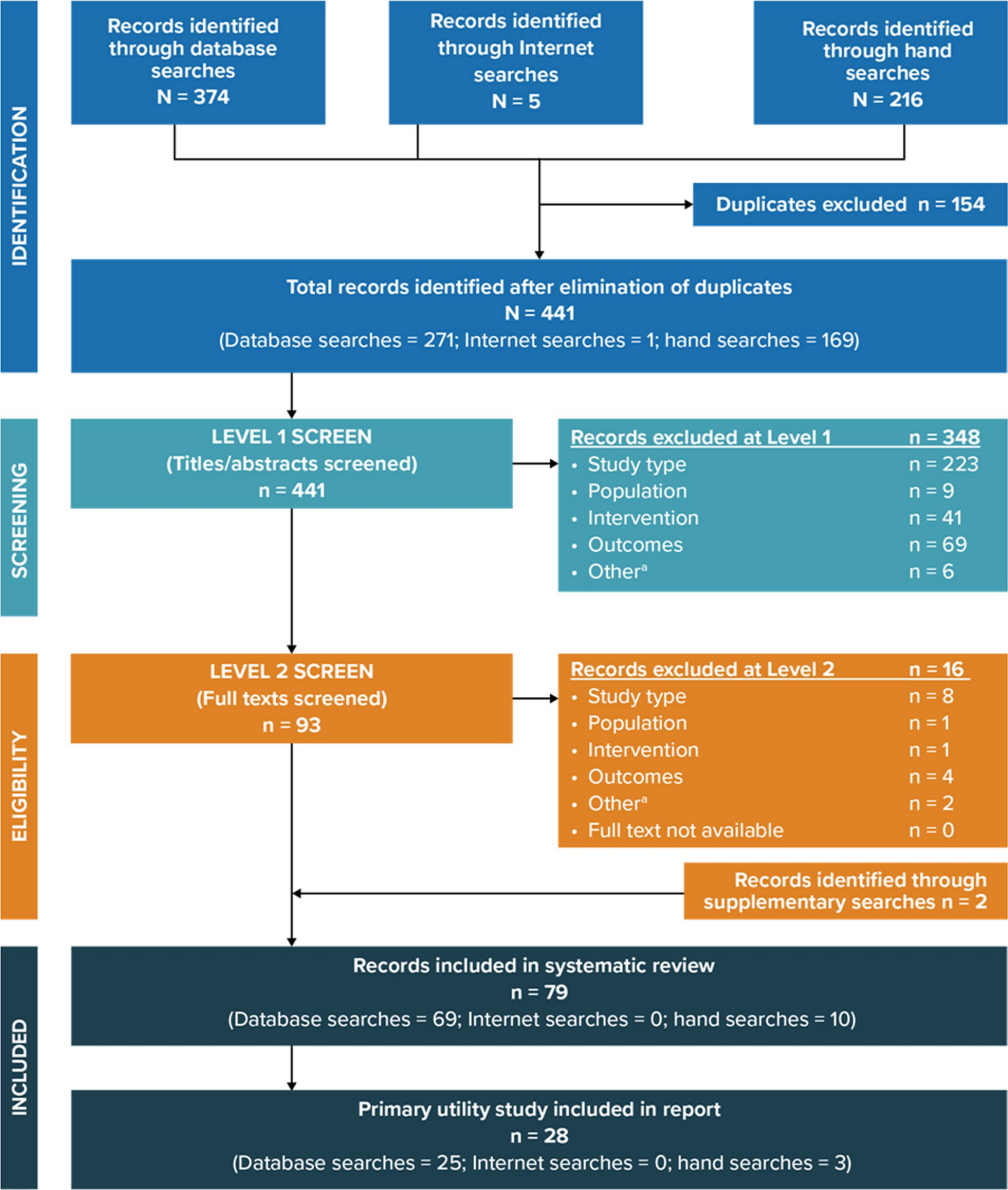
PRISMA

A total of 441 unique records were identified in the literature searches after the removal of duplicates. After the initial screening of titles and abstracts, 93 articles were progressed to full-text review. Of those, 77 articles met the predefined inclusion criteria. An additional two articles were identified through supplemental searches, resulting in a total of 79 articles meeting the predefined inclusion criteria. A total of 28 articles reporting primary utility data for MDD treated with pharmacological interventions were included as the focus of this manuscript, and 51 articles that did not report primary utility data were excluded from this manuscript. The PRISMA (Preferred Reporting Items for Systematic Reviews and Meta-Analyses) diagram [10] presents the study selection process (Fig. 1).

Table 1 presents information about the population, study type and recruitment, and utility data reported in the 28 articles. Articles reporting separate analyses of the same study are grouped together, and studies are organized by geographic region and country. Eleven of the included articles reported analyses from four unique studies in Asia. Of these, seven articles report analyses from a prospective observational study that recruited patients from six East Asian countries [11–17], with utility values first published by Lee et al. [14]. Two articles reported analyses from a prospective observational study in Japan [18, 19], with utility values first published by Kuga et al. [19]. Kim et al. [20] and Husain et al. [21] reported results from a cross-sectional study in South Korea and a randomized controlled trial (RCT) in Pakistan, respectively.

**Table 1:** Summary of included studies

Author, Country	Population description	Study type and recruitment	Summary of reported utilities
Asia			
*Studies linked to primary publication reported by* Lee et al. [14]			
Lee et al. [14], multinational (China, Hong Kong, Korea, Malaysia, Singapore, and Taiwan)	Patients aged ≥ 18 years who presented with new or first episode of MDD (DSM-IV-TR or ICD-10 definition) and were prepared to take ADT. CGI-S score ≥ 4 and at least 2 months free of depressive symptoms before onset of current episode. Age, mean (SD): 45.1 (14.1) years. Females, n (%): 625 (68.8)	Prospective, noninterventional, epidemiological observational study; 909 inpatients and outpatients enrolled from 40 practice sites in 6 East Asian countries from June 14, 2006, to February 15, 2007, and followed for 3 months: China (n = 299), Hong Kong (n = 90), Korea (n = 198), Malaysia (n = 98), Singapore (n = 30), and Taiwan (n = 194)	EQ-5D-3L^a^ utility scores were reported at baseline and at 6 months stratified by the presence of PPS
Ang et al. [11], multinational (China, Hong Kong, Korea, Malaysia, Singapore, and Taiwan)			Baseline EQ-VAS scores stratified by the prescribed intervention (SSRI or SNRI) and change from baseline in EQ-VAS stratified by prescribed intervention and presence of PPS were reported
Lee et al. [13], Korea subanalysis		Subgroup analysis of 198 Korean patients from study conducted in Lee et al. [14]	Baseline EQ-5D-3L^a^ utility scores and change from baseline at 6 months were reported, stratified by the presence of PPS
Chen et al. [12], Taiwan subanalysis		Subgroup analysis of 194 Taiwanese patients from study conducted in Lee et al. [14]	Baseline EQ-5D-3L^a^ utility scores and change from baseline at 3 months were reported, stratified by the presence of PPS and prescribed intervention (SNRI or SSRI)
Li et al. [15], China subanalysis		Subgroup analysis of 299 Chinese patients from study conducted in Lee et al. [14]	EQ-VAS scores were reported at baseline and at 3 months for the overall population, stratified by the presence of PPS and prescribed intervention (SSRI or SNRI)
Novick et al. [17], multinational (China, Hong Kong, Malaysia, Singapore, South Korea, and Taiwan)		Subgroup analysis of 426 patients who started ADT at the baseline visit and had information on adherence during the follow-up period in the study conducted in Lee et al. [14]	EQ-5D-3L^a^ utility scores were reported at baseline and 3 months for patients with clinically reported adherence or nonadherence to ADT
Novick et al. [16], China subanalysis		Subgroup analysis of 300 Chinese patients from study conducted in Lee et al. [14]	EQ-5D-3L^a^ utility and EQ-VAS scores were reported at baseline and at 3 months for the overall population; scores stratified by the presence of PPS were reported at 3 months
*Studies linked to primary publication reported by* Kuga et al. [19]			
Kuga et al. [19], Japan	Patients ≥ 20 years with at least moderate depression (QIDS ≥ 16) and at least moderate PPS (BPI-SF average pain ≥ 3) presenting with an episode of MDD without psychotic traits, defined by the DSM-IV. Age, mean (SD): 42.9 (14.6) years. Female: 51.2%	Prospective, observational, 12-week study conducted at 39 sites, including psychiatry and psychosomatic outpatient/inpatient clinics/hospitals. 523 patients diagnosed by the investigator were recruited, with patient visits occurring between February 13, 2014, and February 26, 2016	Baseline EQ-5D^b^ utility scores and change from baseline at 2, 4, 6, and 12 weeks were reported, stratified by the prescribed intervention (duloxetine or SSRIs)
Kuga et al. [18], Japan		Subgroup analyses of study conducted in Kuga et al. [19]	EQ-5D^b^ utility scores stratified by the prescribed intervention (duloxetine or SSRIs) were reported at week 12 for the following subgroups (at baseline): number of MDEs, BPI-SF average pain score, and HAMD-17 total score
*Unique studies*			
Kim et al. [20], South Korea	Patients aged 19–65 years with a diagnosis of MDD according to the DSM-IV, confirmed by the Mini-International Neuropsychiatric Interview and started on ADT monotherapy either as first-line therapy or as first treatment switch from previous ADT. Age, mean (SD): 45.2 (13.1) years. Female: 74.0%	PERFORM-K was an observational, cross-sectional, multisite study. 343 patients were recruited from 29 psychiatric departments in university or general hospitals throughout South Korea between October 2013 and January 2014	Baseline EQ-5D^b^ utility scores were reported, stratified by severity of depression (MADRS score 0–25, 26–29, 30–34, and 35–60) and severity of perceived cognitive dysfunction (PDQ-D score 0–12, 13–27, 28–43, and 44–80)
Husain et al. [21], Pakistan	Patients aged 18–65 years with DSM-5 MDE that had failed to respond to ≥ 2 ADTs. Age, median (IQR): 40 (30–46) years. Male, n (%): 11 (55)	Multisite, 12-week, double-blind, placebo-controlled pilot trial. 41 patients were recruited from outpatient psychiatric clinics at Abbasi Shaheed Hospital, Karwan-e-Hayat Hospital, Civil Hospital, and the Institute of Behavioural Sciences between October 2014 and March 2016	EQ-VAS scores stratified by the prescribed intervention (minocycline + TAU or placebo + TAU) were reported at baseline and at 12 weeks
Europe			
*Studies linked to primary publication reported by* Montgomery et al. [22]			
Montgomery et al. [22], multinational (Austria, Belgium, Bulgaria, Czech Republic, Estonia, Germany, Italy, Lithuania, Poland, Romania, Russia, Spain, Sweden, and UK)	Patients aged ≥ 18 years and ≤ 75 years with single-episode MDD or recurrent MDD (and a current MDE < 12 months) according to DSM-IV-TR and a MADRS total score ≥ 22 and item 1 score ≥ 3. Eligible patients had an inadequate response to a SSRI/SNRI monotherapy at approved doses for ≥ 6 weeks prior to the screening visit. Age, mean (SD): 47 (12) years. Female, n (%): 195 (77.1)	Double-blind, randomized, flexible-dose, active comparator study including 495 patients recruited from 71 psychiatric inpatient and outpatient settings in 14 countries from January 2012 to December 2012. Patients were recruited via advertisements (in Austria, Germany, Estonia, Russia, Sweden, and UK) or referrals from primary care physicians	EQ-VAS scores at baseline and 8-week change from baseline were reported, stratified by prescribed intervention (vortioxetine or agomelatine)
Papakostas et al. [23], multinational (Austria, Belgium, Bulgaria, Czech Republic, Estonia, Germany, Italy, Lithuania, Poland, Romania, Russia, Spain, Sweden, and UK)		Subgroup analyses of study conducted in Montgomery et al. [22]	EQ-VAS scores at baseline and 8-week change from baseline were reported, stratified by prescribed intervention (vortioxetine or agomelatine) and previous treatment (SSRI or SNRI)
Studies linked to primary publication reported in Garcia-Cebrian et al. [24]			
Garcia-Cebrian et al. [24], multinational (Austria, Belgium, France, Germany, Ireland, Italy, Netherlands, Norway, Portugal, Sweden, Switzerland, and UK)	Patients aged ≥ 18 years with clinical diagnosis of depression by their physician and about to start ADT for a first or subsequent episode of depression. Age, mean (SD): 46.8 (14.7) years Female, 68.2%. Duration of depressive illness, mean (SD), 8.5 (10.4) years, Duration of current episode, mean (SD): 13.6 (16.5) weeks	European, prospective, observational study (FINDER). 3515 patients presenting during the normal course of care were enrolled by 437 primary care physicians or specialists (mostly psychiatrists) from 12 countries between May 2004 and September 2005. Data were collected at baseline, 3 months, and 6 months during routine visits	EQ-5D-3L^a^ utility and EQ-VAS scores were reported at baseline
Reed et al. [25], multinational (Austria, Belgium, France, Germany, Ireland, Italy, Netherlands, Norway, Portugal, Sweden, Switzerland, and UK)		Subsequent analysis of study reported in Garcia-Cebrian et al. [24]	EQ-5D-3L^a^ utility and EQ-VAS scores were reported at baseline, 3 months, and 6 months for patients receiving ADT
*Unique studies*			
Kuyken et al. [27], UK	Patients aged ≥ 18 years with recurrent MDD in full or partial remission according to DSM-IV currently on a therapeutic dose of ADT. Patients had ≥ 3 previous MDEs in which depression was the primary disorder and was not secondary to substance abuse, bereavement, or a general medical condition MBCT-TS: Female, n (%): 151 (71) Age, mean (SD): 50 (12) years ADM: Female, n (%): 174 (82) Age, mean (SD): 49 (13) years	Economic evaluation alongside a clinical trial. Two-arm, multicenter, single-blind superiority trial. Patients identified through physician and telephone assessment. 95 patients recruited from primary care in urban and rural settings in 4 UK centers: Bristol, Exeter and East Devon, North and Mid Devon, and South Devon	EQ-5D-3L utility scores were reported at baseline and at 1, 9, 12, 18, and 24 months by prescribed intervention (MBCT-TS or maintenance ADM)
Serfaty et al. [29], UK	Patients ≥ 65 years with a primary diagnosis of depressive disorder made by the researcher who administered the Geriatric Mental State and History and Etiology Schedule and a BDI-II score of ≥ 14. Age, mean (SD): 75 (7.1) years. Female, n (%): 50 (74.6)	Single-blind, randomized controlled trial that took place between April 2004 and September 2007. Patients were recruited by self-referral, primary care referral, and by database searches. Patients who scored ≥ 5 on GDS-15 were offered a further interview to see whether they satisfied entry criteria. 204 patients were randomized	EQ-5D-3L utility scores were reported at baseline, 4, and 10 months by prescribed intervention (CBT + TAU, TC + TAU, or TAU)
Morriss et al. [28], UK	Patients ≥ 18 years with persistent moderate or severe primary unipolar depression with a current MDE (DSM-IV), met 5 of 9 NICE criteria for symptoms of moderate depression, had HAMD-17 ≥ 16, and had GAF ≤ 60. Age, mean (SD): 46 (11.3) years. Female n (%): 60 (64%)	Economic evaluation alongside a clinical trial. Patients currently under the care of a secondary care mental health team were recruited from 3 sites (Cambridge, Derby, and Nottingham) for a multicenter, single-blind, patient-level, parallel, randomized controlled trial. 187 patients were randomized	EQ-5D-3L utility scores were reported at baseline and at 6, 12, and 18 months by prescribed intervention (TAU or SDS)
Sapin et al. [30], France	Patients aged 18–92 years who consulted a primary care physician for a new episode of MDD according to DSM-IV and had not previously received ADT. Age, mean (SD): 44.2 (14.1) years. Sex ratio (males/females): 0.4. MADRS score, mean (SD): 32.7 (7.7)	National, multicenter, prospective, noncomparative cohort study with a scheduled follow-up period of 2 months. 250 outpatients were enrolled by 95 physicians between May and November 2002	EQ-5D-3L utility scores were reported for the total population at baseline, and at baseline, 4 weeks and 8 weeks, stratified by clinical response, (responder remitters, responder nonremitters, and nonresponders) according to changes in MADRS scores
Fernandez et al. [26], multinational (Europe)	Patients aged 18–85 years with moderate to severe MDD (DSM-IV), without suicidal tendencies, and a MADRS total score ≥ 18 at screening 1 week before start of ADT and at start of ADT Venlafaxine: Female n (%): 89 (71.2) Age, mean (SD): 46.5 (13.5) years Escitalopram: Female n (%): 95 (75.4) Age, mean (SD): 48.4 (14.7) years	Economic evaluation alongside a clinical trial reported by Montgomery et al. [22]. 293 outpatients recruited for a randomized, double-blind, flexible-dose, clinical trial across 8 European countries	EQ-5D-3L^a^ utility scores were reported by prescribed intervention (escitalopram or venlafaxine) at baseline and 8 weeks
Saragoussi et al. [31], multinational (France, Germany, Spain, Sweden, and UK)	Patients aged 18–65 years with MDD according to DSM-IV initiating or switching to an ADT. Age, mean (SD): 44.3 (12.0) years. Female: (73.2%)	2-years, multicenter, prospective, noninterventional cohort study (PERFORM) that enrolled 1159 outpatients by either a primary care physician or a psychiatrist at 194 sites in France, Germany, Spain, Sweden, and the UK	EQ-5D-3L^a^ scores were reported at baseline, and at 2, 6, 12, 18, and 24 months for patients receiving antidepressant monotherapy or undergoing first switch of ADT
The Americas			
Soares et al. [32], multinational (Argentina, Chile, Colombia, Mexico, and US)	Postmenopausal women aged 40–70 years with a primary diagnosis of MDD based on MINI and DSM-IV criteria, and depressive symptoms for ≥ 30 d before screening visit and a MADRS total score of ≥ 22 at screening and baseline, with a ≤ 5-point improvement from screening to baseline. Age, mean (SD): 55 (6) years in escitalopram double blind/desvenlafaxine OL cohort and 54 (6) years in desvenlafaxine DB/desvenlafaxine OL	Randomized, phase 3b, parallel group, comparator-controlled, multicenter study with an 8-week, double-blind acute phase followed by a 2-arm, 6-month extension phase conducted from December 2006 to September 2008. 607 patients were enrolled from 72 sites across Argentina, Chile, Colombia, Mexico, and the US and randomized in the acute phase. 129 patients entered the open-label extension phase	Change in EQ-5D-3L^a^ utility scores from acute-phase baseline and extension-phase baseline were reported
Revicki and Wood [33], multinational (Canada and US)	Patients aged 18–65 years with DSM-III-R diagnosis of MDD based on clinician interview using the SCID and completed at least 8 weeks of ADT or had completed a regimen within the last 2 months. Patients with dysthymia were allowed. N = 70. Age, mean (SD): 42 (11) years; male: 23%; married: 48%; HAMD-17 score, mean (SD): 11.65 (8.2)	Prospective observational study, 70 outpatients enrolled from a university family practice clinic in Toronto or a community-based primary care practice in San Diego; Canada (n = 40) and US (n = 30)	Standard gamble utility scores were reported for the total population and for a series of hypothetical health states based on HAMD-17 depression severity: patients with severe depression, untreated; patients with moderate depression, mild depression, and in remission, all stratified by intervention received (nefazodone, fluoxetine, or imipramine); patients in remission receiving no treatment
Multiregional			
*Studies linked to primary publication reported by* Duenas et al. [34]			
Duenas et al. [34], multinational (China, Hong Kong, Malaysia, Philippines, Taiwan, Thailand, Singapore, Saudi Arabia, United Arab Emirates, Mexico, Israel, and Austria)	Outpatients aged ≥ 18 years with a primary diagnosis of MDD (excluding treatment-resistant depression) according to ICD-10 or DSM-IV-TR criteria who were at least moderately depressed (CGI-S score ≥ 4), presenting with an episode of MDD and initiating, or switching to, any available SSRI or SNRI. Patients were sexually active without sexual dysfunction (sexual dysfunction defined as ASEX total score ≥ 19, ASEX score ≥ 5 on any item, or ASEX score ≥ 4 on any 3 items). Age, mean (SD): 38.2 (10.3), years. Female, n (%): 319 (51.8)	6-month, prospective, observational study. 1659 patients were enrolled from 89 sites in 12 countries between November 15, 2007, and November 28, 2011; China (n = 199), Hong Kong (n = 17), Malaysia (n = 33), Philippines (n = 110), Taiwan (n = 180), Thailand (n = 8), Singapore (n = 2), Saudi Arabia (n = 168), United Arab Emirates (n = 116), Mexico (n = 581), Israel (n = 9), and Austria (n = 42)	Change from baseline in EQ-5D-3L^a^ utility scores and EQ-VAS at 8 weeks were reported by prescribed intervention (duloxetine or SSRI) for all patients and stratified for patients with and without TESD
Duenas et al. [35], multinational (China, Hong Kong, Malaysia, Philippines, Taiwan, Thailand, Singapore, Saudi Arabia, United Arab Emirates, Mexico, Israel, and Austria)			Change from baseline in EQ-5D-3L^a^ utility scores and EQ-VAS at 6 months were reported by prescribed intervention (duloxetine or SSRI) for all patients and stratified for patients with and without TESD
Hong et al. [37], multinational (China, Hong Kong, Malaysia, Philippines, Taiwan, Thailand, Singapore, Saudi Arabia, United Arab Emirates, Mexico, Israel, and Austria)		Post-hoc analysis of the study reported in Duenas et al. [34]	EQ-5D-3L^a^ utility scores and EQ-VAS scores were reported at baseline, 8 weeks, 16 weeks, and 24 weeks for the total population and PPS + and PPS − populations, stratified by the prescribed intervention (duloxetine or SSRI)
Hong et al. [36], East Asia subanalysis (China, Hong Kong, Malaysia, the Philippines, Taiwan, Thailand, and Singapore)		Post-hoc subgroup analysis of 587 East Asia patients from study conducted in Hong et al. [37]; China (n = 205), Hong Kong (n = 18), Malaysia (n = 33), the Philippines (n = 113), Taiwan (n = 199), Thailand (n = 17), and Singapore (n = 2)	EQ-5D-3L^a^ utility scores and EQ-VAS scores were reported at baseline, stratified by the prescribed intervention (duloxetine or SSRI), and at 24 weeks, stratified by the prescribed intervention and presence of PPS
*Unique studies*			
Florea et al. [38], multinational	MDD patients aged ≥ 18 years who received the approved doses of vortioxetine 5, 10, 15, and 20 mg/d	Across-study comparison of HRQoL included in 5 short-term (6–8 week) studies (NCT00672958, NCT00735709, NCT00635219, NCT00839423, NCT01140906) and 1 dedicated study in elderly patients (NCT00811252)	EQ-5D-3L^a^ health state score change from baseline at 6 weeks for different doses of vortioxetine versus placebo

*ADM* Antidepressant Medication, *ADT* Antidepressant Treatment, *ASEX* Arizona Sexual Experience Scale, *BDI-II* Beck Depression Inventory-II, *BPI-SF* Brief Pain Inventory (Short Form), *CBT* Cognitive-Behavioral Therapy, *CGI-S* Clinical Global Impressions-Severity of Illness, *DSM-5* Diagnostic and Statistical Manual of Mental Disorders, Fifth Edition, *DSM-III-R* Diagnostic and Statistical Manual of Mental Disorders, Third Edition, Revised, *DSM-IV* Diagnostic and Statistical Manual of Mental Disorders, Fourth Edition, *DSM-IV-TR* Diagnostic and Statistical Manual of Mental Disorders, Fourth Edition, Text Revision, *EQ-5D-3L* 3-Level EQ-5D, *EQ-VAS EQ* Visual Analogue Scale, *GAF* Global Assessment of Functioning, *GDS-15* Geriatric Depression Scale-15, *HAMD-17* Hamilton Rating Scale for Depression-17, *HRQoL* Health-Related Quality of Life, *ICD-10* International Classification of Diseases, 10th Revision, *ID* Identifier, *IQR* Interquartile Range, *MADRS* Montgomery-Åsberg Depression Rating Scale, *MBCT-TS* Mindfulness-Based Cognitive Therapy with Support to Taper, *MDD* Major Depressive Disorder, *MDE* Major Depressive Episode, *MINI* Mini-International Neuropsychiatric Interview, *NCT* National Clinical Trial, *NICE* National Institute for Health and Care Excellence, *OL* Open Label, *PDQ-D* Perceived Deficits Questionnaire–Depression, *PPS* Painful Physical Symptoms, *QIDS* Quick Inventory of Depressive Symptomatology, *SCID* Structured Clinical Interview for DSM-IV, *SD* Standard Deviation, *SDS* Specialist Depression Services, *SNRI* Serotonin-Norepinephrine Reuptake Inhibitor, *SSRI* Selective Serotonin Reuptake Inhibitor, *TAU* Treatment As Usual, *TC* Talking Control, *TESD* Treatment-Emergent Sexual Dysfunction, *UK* United Kingdom, *US* United States

^a^EQ-5D-3L was not explicitly stated in the study, but was deduced, either from the date of the study or from the date of references to EQ-5D methodology (the EQ-5D-5L was introduced after 2009)

^b^The use of EQ-5D-3L or EQ-5D-5L was not stated and nor could it be deduced based on the date of the study or references

Ten of the included articles reported analyses from eight unique studies in Europe. Of these, two articles reported analyses from an RCT that recruited patients from 14 countries [22, 23], with utility values first published by Montgomery et al. [22]. Garcia-Cebrian et al. [24] reported results from a prospective observational study that recruited patients from 12 countries, and Reed et al. [25] reported a subsequent analysis of this study. Of the remaining six studies in Europe, three are economic evaluations alongside clinical trials [26–28], one is an RCT [29], and two are prospective observational studies [30, 31]. Of these, the studies reported by Kuyken et al. [27], Serfaty et al. [29], and Morriss et al. [28] were conducted in the United Kingdom (UK), the study reported by Sapin et al. [30] was in France, the study reported by Fernandez et al. [26] was in eight European countries, and the study reported by Saragoussi et al. [31] was in five European countries.

Two of the included articles reported unique studies in the Americas. Soares et al. [32] reported results from an RCT conducted in five countries across North and South America. Revicki and Wood [33] reported results from a prospective observational study conducted in Canada and the United States (US).

Five of the included articles reported analyses of two unique studies in more than one region. Duenas et al. [34] first reported results from a prospective observational study that recruited patients from 12 countries across Asia, Europe, and the Americas. Three of the articles reported subsequent analyses of this study [35–37]. Florea et al. [38] reported results from an across-study comparison of six multinational clinical trials of vortioxetine.

Overall, 16 unique studies were reported in the 28 included articles. Twenty-two of the articles (13 unique studies) reported utility estimates elicited indirectly; all of these used the EQ-5D. Of these, 19 articles (11 unique studies) used the EQ-5D-3L, and use of the EQ-5D-3L or EQ-5D-5L could not be determined in three articles (2 unique studies). Thirteen articles (6 unique studies) [11, 12, 15–17, 21–25, 35–37] reported utility estimates elicited directly, of which 12 used a VAS (EQ-VAS) and one used the SG technique [33]. Seven articles (3 unique studies) reported utility estimates elicited using both the EQ-5D and EQ-VAS.

Table 2 presents a summary of the HSUVs reported in the included studies. Information about the health state and clinical features, MDEs and prior therapy of the population, and the interventions are presented for each HSUV, along with the instrument used. Reported utility estimates where a specific health state could not be ascertained (for example, aggregated utility estimates for a study population at a time point where patients had different levels of treatment response) and indirect instrument scores where utility tariffs had not been applied (for example, EQ-5D domain scores) were excluded. Six articles did not report relevant HSUVs and were excluded from Table 2 [19, 32, 34–36, 38]. Reed et al. [25] reported the same HSUV as that in the primary analysis by Garcia-Cebrian et al. [24]. A quality assessment of the studies reporting relevant HSUVs (using criteria from Papaioannou et al. [39]) is presented in Additional file 6.

**Table 2:** Summary of health state utility values from included studies

Author, Country	Health state	Clinical stratification	MDE	Prior therapy	Intervention	Utility estimate (SD)^a^	Instrument
*Asia*							
Lee et al. [14], multinational	MDD	PPS +	New or first episode	NR	Starting new ADT	0.37 (0.01)	EQ-5D-3L^b^
		PPS −				0.61 (0.02)	
Ang et al. [11], multinational	MDD	Overall	New or first episode	NR	Starting an SSRI	0.497 (0.1945)	EQ-VAS
					Starting an SNRI	0.437 (0.2076)	
Lee et al. [13], Korea	MDD	PPS +	New or first episode	NR	Starting new ADT	0.2 (0.36)	EQ-5D-3L^b^ (UK tariff; Korean population has not been validated)
		PPS −				0.5 (0.30)	
Chen et al. [12], Taiwan	MDD	Overall	New or first episode	NR	Starting new ADT	0.481 (0.1978)	EQ-VAS
					Starting an SSRI	0.488 (0.2017)	
					Starting an SNRI	0.463 (0.1874)	
		PPS +			Starting new ADT	0.429 (0.1826)	
		PPS −			Starting new ADT	0.598 (0.1821)	
Li et al. [15], China	MDD	Overall	New or first episode	NR	Starting an SNRI	0.4553 (0.2364)	EQ-VAS
					Starting an SSRI	0.4717 (0.1897)	
					Starting new ADT	0.4581 (0.2057)	
		PPS +				0.3848 (0.2238)	
		PPS −				0.4957 (0.1854)	
Novick et al. [17], multinational	MDD	Adherent	New or first episode	NR	Starting new ADT	0.46 (0.34)	EQ-5D-3L^b^ (UK tariff)
		Nonadherent				0.37 (0.36)	
		Adherent				0.4536 (0.1962)	EQ-VAS
		Nonadherent				0.4884 (0.2010)	
Novick et al. [16], China	MDD	Overall	New or first episode	NR	Starting new ADT	0.52 (0.31)	EQ-5D-3L^b^ (UK tariff)
						0.455 (0.209)	EQ-VAS
Kuga et al. [19], Japan	MDD	PPS +	NR	No ADT for at least 4 weeks	Starting duloxetine	0.5055 (0.1504)	EQ-5D^c^
					Starting an SSRI	0.5016 (0.1602)	
Kim et al. [20], South Korea	MDD	Overall	New or first episode (59.6% were newly diagnosed)	22.8% had received prior ADT	Receiving ADT	0.726 (0.192)	EQ-5D^c^
	Very severe MDD	MADRS 35–60				0.615 (0.209)	
	Severe MDD	MADRS 30–34				0.718 (0.186)	
	Moderate MDD	MADRS 26–29				0.746 (0.145)	
	Remission or mild MDD	MADRS 0–25				0.806 (0.166)	
Husain et al. [21], Pakistan	Treatment-resistant MDD	Overall	Current episode ≥ 4 weeks	Current episode has failed to remit with ≥ 2 ADTs (including current ADT received for ≥ 4 weeks)	Starting placebo (with current ADT)	0.44 (0.191)	EQ-VAS
					Starting minocycline (with current ADT)	0.424 (0.167)	
*Europe*							
Montgomery et al. [22], multinational	MDD	Overall	Single or recurrent episode < 12 months duration	Inadequate response to ADT for ≥ 6 weeks prior to study screening	Starting agomelatine	0.468 (0.194)	EQ-VAS
					Starting vortioxetine	0.455 (0.183)	
Papakostas et al. [23], multinational	MDD with nonresponse or partial response to 1 ADT	Overall	Single or recurrent episode; current episode < 12 months duration	Inadequate response to SSRI ≥ 6 weeks prior to study screening	Starting agomelatine	0.470 (0.198)	EQ-VAS
					Starting vortioxetine	0.449 (0.185)	
				Inadequate response to SNRI ≥ 6 weeks prior to study screening	Starting agomelatine	0.464 (0.185)	
					Starting vortioxetine	0.470 (0.176)	
Garcia-Cebrian et al. [24], and Reed et al. [25], multinational	MDD	Overall	New or first episode	NR	Starting new ADT	0.44 (0.31)	EQ-5D-3L^b^
Kuyken et al. [27], UK	Recurrent MDD in full or partial remission	Overall	3 or more previous episodes	Included patients who “were on a therapeutic dose of ADM in line with the British National Formulary (BNF) 48 and NICE guidance”	Starting m-ADT	0.778 (0.211)	EQ-5D-3L
					Starting MBCT-TS	0.760 (0.268)	
Serfaty et al. [29], UK	MDD	Overall	77% of the total population had previous episode of depression	If taking an ADT, on a stable dose for at least 8 weeks prior to the study	ADT	0.46 (0.29)	EQ-5D
					ADT + CBT	0.50 (0.32)	
					ADT + TC	0.52 (0.31)	
Morriss et al. [28], UK	MDD	Overall	Current episode	Offered or received direct and continuous care in the preceding 6 months	Starting TAU	0.337 (0.343)	EQ-5D-3L
					Starting SDS	0.361 (0.329)	
Sapin et al. [30], France	MDD	Overall	New episode	No prior ADT	Starting ADT	0.33 (0.25)	EQ-5D-3L^b^
	Responders remitters	MADRS ≤ 12			Received ADT for 8 weeks	0.85 (0.13)	
	Responders nonremitter	MADRS decrease > 50%				0.72 (0.20)	
	Nonresponders	MADRS decrease < 50%				0.58 (0.28)	
Fernandez et al. [26], multinational	MDD	Overall	New episode	No prior ADT	Starting escitalopram	0.52 (0.3)	EQ-5D-3L^b^ (UK tariff)
					Starting venlafaxine	0.54 (0.3)	
Saragoussi et al. [31], multinational	MDD	Overall	Current episode	21.3% switching ADT and 78.7% no prior ADT	Starting new ADT	0.5 (0.3)	EQ-5D-3L^b^ (UK tariff)
*The Americas*							
Revicki and Wood [33], Canada and US	MDD	Overall	Current episode	Receiving current ADT or had completed an ADT regimen within the last 2 months prior to the study	Receiving ADT	0.74 (0.22)	Standard gamble (patients)
	Severe MDD				Untreated	0.30 (0.28)	
	Moderate MDD				Nefazodone	0.63 (0.23)	
					Fluoxetine	0.63 (0.19)	
					Imipramine	0.55 (0.03)	
	Mild MDD				Nefazodone	0.73 (0.21)	
					Fluoxetine	0.70 (0.20)	
					Imipramine	0.64 (0.20)	
	Remission (maintenance treatment)				Nefazodone	0.83 (0.13)	
					Fluoxetine	0.80 (0.15)	
					Imipramine	0.72 (0.17)	
	Remission (off treatment)				Untreated	0.86 (0.16)	
*Multiregional*							
Hong et al. [37], multinational	MDD	Overall	Current episode	Initiating or switching ADT with up to 1 previous inadequate response to ADT; no ADT received 1 week prior to study	Starting duloxetine	0.46 (0.32)	EQ-5D-3L^b^ (UK tariff)
					Starting an SSRI	0.47 (0.33)	
		PPS +			Starting duloxetine	0.37 (0.33)	
					Starting an SSRI	0.40 (0.34)	
		PPS −			Starting duloxetine	0.58 (0.26)	
					Starting an SSRI	0.54 (0.31)	
		Overall			Starting duloxetine	0.4339 (0.2436)	EQ-VAS
					Starting an SSRI	0.4299 (0.2694)	
		PPS +			Starting duloxetine	0.4077 (0.2423)	
					Starting an SSRI	0.3948 (0.2725)	
		PPS −			Starting duloxetine	0.4707 (0.2411)	
					Starting an SSRI	0.4602 (0.2636)	

*ADM* Antidepressant Medication, *ADT* Antidepressant Treatment, *CBT* Cognitive-Behavioral Therapy, *EQ-5D-3L* 3-Level EQ-5D, *EQ-VAS EQ* Visual Analogue Scale, *MADRS* Montgomery-Åsberg Depression Rating Scale, *MBCT-TS* Mindfulness-Based Cognitive Therapy with Support to Taper, *MDD* Major Depressive Disorder, *MDE* Major Depressive Episode, *NICE* National Institute for Health and Care Excellence, *NR* Not Reported, *PPS* Painful Physical Symptoms, *SD* Standard Deviation, *SDS* Specialist Depression Services, *SNRI* Serotonin-Norepinephrine Reuptake Inhibitor, *SSRI* Selective Serotonin Reuptake Inhibitor, *TAU* Treatment As Usual, *TC* Talking Control, *UK* United Kingdom, *US* United States

^a^EQ-VAS scores were converted from a 0–100 scale to a 0–1 scale

^b^EQ-5D-3L was not explicitly stated in the study, but was deduced, either from the date of the study or from the date of references to EQ-5D methodology (the EQ-5D-5L was introduced after 2009)

^c^The use of EQ-5D-3L or EQ-5D-5L was not stated, nor could it be deduced based on the date of the study or references

Most of the articles included in Table 2 reported HSUVs at baseline, with the MDD health state defined by the study population entry criteria. Many of the articles reported utility estimates at subsequent time points where a specific health state could not be ascertained. Considerable heterogeneity in patient characteristics was found between the studies, and a wide range of utility values were reported. The baseline HSUVs can be differentiated based on key features of the study populations, including severity of MDD, current MDE status (i.e., presenting with a first or new episode, or within an existing episode), lines of prior therapy, and presence of comorbidities [analyses of the studies first reported by Lee et al. [14] and Duenas et al. [34] stratified baseline estimates by the presence of painful physical symptoms (PPS)]. Treatments under investigation were also heterogenous; some articles specified which treatments were investigated, while others stated that investigations included only SSRIs or treatment as usual, which made comparison between the studies difficult. Several articles reported HSUVs at baseline for patients presenting with a first or recurrent MDE who were about to start a new treatment. The utility estimates ranged from 0.33 [30] to 0.544 [17] at baseline. The range widened when estimates stratified by the presence of PPS were included; the lowest estimate for patients with PPS was 0.20 [14], and the highest estimate for patients without PPS was 0.61 [13]. Several articles reported HSUVs at baseline for patients with an existing MDE who inadequately responded to treatment and were about to switch therapy, with utility estimates ranging from 0.337 [28] to 0.449 [23].

Three studies reported utility estimates for health states defined by specific clinical thresholds [20, 30, 33]. Kim et al. [20] reported EQ-5D HSUVs for South Korean patients receiving ADT during the usual course of care stratified by disease severity defined by MADRS score thresholds (very severe, severe, moderate, mild, or remission). The HSUVs increased progressively through disease severities, from 0.615 for patients with very severe MDD (MADRS score: 35–60) to 0.806 for patients with mild MDD or remission (MADRS score: 0–25). Sapin et al. [30] reported EQ-5D HSUVs for French patients who had received first-line ADT for 8 weeks, stratified by treatment response (responder remitters, responder nonremitters, nonresponders). Remission was defined by a MADRS score threshold, whereas *responder nonremitter* and *no response* were defined by thresholds for percentage change in MADRS score. This was the only study to use thresholds based on percentage changes in clinical scores and to report HSUVs at a specific time point after study entry. The HSUVs increased from 0.33 for patients with MDD at baseline before treatment to 0.58 for patients with no response (< 50% decrease from baseline in MADRS score), 0.72 for patients with nonremitting response (≥ 50% decrease from baseline in MADRS score), and 0.85 for patients in remission (MADRS score ≤ 12). Revicki and Wood [33] reported directly elicited HSUVs for Canadian and US patients who were receiving or who had recently completed an ADT regimen (nefazodone, fluoxetine, or imipramine) within the last 2 months prior to study entry, stratified by disease severity (severe, moderate, mild, remission on or off treatment) defined by HAMD score thresholds (the thresholds were not reported). The HSUVs were stratified by treatment received and increased progressively through disease severities, from 0.30 for patients with severe MDD (untreated) to 0.86 for patients in remission (off treatment). Kuyken et al. [27] reported EQ-5D HSUVs for UK patients with recurrent MDD with three or more previous MDEs in full or partial remission. However, remission was defined using the DSM-IV at study entry rather than by a clinical measure used within the trial.

Revicki and Wood [33] was the only study to report disutility estimates associated with treatment side effects (Table 3). Patients had been treated previously with fluoxetine, imipramine, nefazodone, or a combination of treatments; however, the study did not report adverse events by the different treatments. Disutilities were reported for several key adverse events associated with the ADTs, but the study was published in 1998 and may not represent current practice. Disutilities were calculated as the difference between mean SG utilities elicited directly from patients with and without specific adverse events. The mean differences ranged from 0.01 for nausea or dry mouth to 0.12 for nervousness and light-headedness/dizziness (Table 3), with the latter being the only statistically significant difference (*P* = 0.030).

**Table 3:** Summary of adverse event disutilities from included studies

Author (year), Country	Population	Adverse event	Disutility estimate	Instrument
Revicki and Wood [33], Canada and United States	Patients with MDD who had completed at least 8 weeks of ADT or had completed an ADT regimen within the last 2 months prior to the study	Sedation	0.06^a^	Standard gamble (patients)
		Nervousness	0.12	
		Headache	0.09^a^	
		Constipation	0.05^a^	
		Tension	0.035^a^	
		Lightheaded/faint	0.12	
		Dry mouth	0.01	
		Nausea	0.01	

*ADT* Antidepressant Treatment, MDD Major Depressive Disorder

^a^Disutilities were calculated

## Discussion

Health state utility values in MDD are required for use in cost-utility analyses for new treatments. Health states experienced by patients with MDD include different severities of depression (i.e., mild, moderate, and severe), different levels of treatment response (i.e., remission, response, and no response), a return to normal health (i.e., recovery), and disease progression (i.e., relapse and recurrence). This systematic literature review identified 79 articles reporting utility estimates for patients with MDD receiving pharmacological treatment, of which 28 reported primary utility data across a range of health states that can be used in economic models.

### HSUVs

Overall, a range of HSUVs were identified that can be used as parameters in a cost-utility model. However, the values were predominantly captured at study baseline, with health states defined by study entry criteria rather than specific clinical thresholds. Many of the studies, particularly RCTs, did capture utility estimates at other time points, but specific health states could not be determined. Such estimates may be suitable to include in simple economic analyses mirroring clinical trials but not for models with distinct health states requiring HSUVs. Three studies did report HSUVs for different depression severity levels that could be used for models with health states defined by corresponding clinical thresholds. Only one study (Sapin et al. [30]) reported HSUVs for a response health state, which was defined by a percentage change in clinical score. While this definition is often used in clinical practice, response could be defined by using a specific clinical threshold within an economic model that allows use of alternative HSUVs based on depression severity. Similarly, no HSUVs were identified specifically for a relapse health state, but relapse could be defined by using a specific clinical threshold within an economic model. For a recovery health state (i.e., a return to normal health), general population utility values could be used. While HSUVs are available to populate a model, the evidence base is limited, and it is important to select values that align with clinical thresholds used within a model.

The pharmacological treatments that were captured by the systematic literature review included SSRIs (including escitalopram and fluoxetine), SNRIs (including duloxetine and venlafaxine), TCAs, selective serotonin receptor antagonists, and serotonin antagonist and reuptake inhibitors. Some of the studies identified did not specify the treatment, instead listing SSRIs, SNRIs, or physician’s choice. There is a data gap of utility estimates available for augmentation agents used alongside ADT, such as lithium and atypical antipsychotics. Future studies could be performed to elicit HSUVs for augmentation agents commonly used for treatment-resistant MDD. This would allow the impact of adverse events associated with augmentation agents on HRQoL to be more easily evaluated. If treatment-independent HSUVs are used in a cost-utility model, it is important to select values from studies with a population that corresponds to that of the intervention being evaluated in an economic analysis.

### Disutility estimates

Revicki and Wood [33] was the only study to report disutility estimates for treatment-related adverse events.

Future studies could be conducted to elicit disutilities for a comprehensive set of adverse events associated with current MDD treatments. It is important that the impact of HRQoL in models that do not use treatment-specific HSUVs be accurately captured, particularly when comparisons are made between treatments with similar efficacy that can be differentiated by their side-effect profiles.

### Suitability for HTA

Utility estimates elicited indirectly using generic, preference-based instruments are preferred by most HTA bodies, and the EQ-5D is often specifically recommended in pharmacoeconomic guidelines [6]. The majority of the studies (13) reported utility estimates elicited indirectly using the EQ-5D. Of these, 11 studies used the EQ-5D-3L; use of the three-level or five-level version could not be determined in 2 studies. Seven articles stated the tariff used for valuation; all used the UK tariff. Three studies reported utility estimates elicited using both the EQ-5D and EQ-VAS [16, 17, 37]; of these, the EQ-5D utilities are preferred by most HTA bodies [6]. In total, six studies reported utility estimates elicited directly from patients; five of these used the EQ-VAS [11, 12, 15, 22, 23], and one used the SG technique [33]. Utility estimates elicited directly using choice-based tasks such as SG are generally considered methodologically superior to those elicited using rating tasks such as a VAS because they incorporate additional information about individual risk attitude. Moreover, rating tasks are prone to scaling biases [7]; as such, the identified utility estimates elicited using EQ-VAS may be less preferable to HTA bodies than the other directly elicited estimates using SG and indirectly elicited EQ-5D estimates. The relevance of studies should be assessed in accordance with local HTA requirements before data are used in a cost-utility model.

Collection of EQ-5D data is recommended in future trials for new treatments that will undergo reimbursement submissions to HTA bodies. Moreover, it is important to elicit HSUVs for the core health states that will be used within a cost-utility model, such as response, remission, relapse, and no response, using appropriate clinical thresholds. Additional utility studies could be performed to elicit utility estimates for health states and adverse events needed within a model, for which there is a paucity of data.

### Study limitations

The primary aim of the study was to identify utility estimates that can be used to populate future economic models for new pharmacological treatments in MDD. Therefore, the literature review focused on studies of patients with MDD receiving pharmacological treatment. However, nonpharmacological interventions such as cognitive-behavioral therapy and transcranial magnetic stimulation are also used in the treatment of MDD, particularly for patients with more severe or treatment-resistant MDD. Studies in patients with MDD receiving nonpharmacological treatments without pharmacological treatment were outside the scope of this review. Additionally, screening of articles was conducted by a single researcher with a random 20% quality check of studies performed by a second researcher, which means that there is a small chance that relevant studies were missed.

## Conclusions

This study systematically identified published HSUVs and disutilities for patients with MDD receiving pharmacological treatment that can be used as parameters within future economic evaluations. Health state utility values, elicited using methods accepted by HTA bodies, are available for key MDD health states defined by clinical thresholds. However, there is a limited evidence base from studies with heterogenous populations and clinical definitions. It is important to select HSUVs that are appropriate for the intervention being evaluated and that align with clinical health state definitions used within a model. Only one study reported disutilities associated with adverse events. It is recommended to elicit HSUVs in clinical trials for new treatments that may undergo reimbursement submissions to HTA bodies and to conduct utility studies where data gaps exist.

## Supplementary Information

**Additional file 1:** PubMed literature search strategy.

**Additional file 2:** Embase literature search strategy.

**Additional file 3:** Cochrane literature search strategy.

**Additional file 4:** EconLit literature search strategy.

**Additional file 5:** Study inclusion and exclusion criteria.

**Additional file 6:** Quality assessment of studies.

## Data Availability

All data generated or analyzed during this study are included in this published article and its supplementary information files.

## Abbreviations

ADM: Antidepressant Medication
ADT: Antidepressant Treatment
ASEX: Arizona Sexual Experience Scale
BDI-II: Beck Depression Inventory-II
BPI-SF: Brief Pain Inventory (Short Form)
CBT: Cognitive-Behavioral Therapy
CGI-S: Clinical Global Impressions-Severity of Illness
DSM-5: Diagnostic and Statistical Manual of Mental Disorders, Fifth Edition
DSM-III-R: Diagnostic and Statistical Manual of Mental Disorders, Third Edition, Revised
DSM-IV: Diagnostic and Statistical Manual of Mental Disorders, Fourth Edition
DSM-IV-TR: Diagnostic and Statistical Manual of Mental Disorders, Fourth Edition, Text Revision
DSM-V: Diagnostic and Statistical Manual of Mental Disorders, Fifth Edition
EQ-5D-3L: 3-Level EQ-5D
EQ-5D-5L: 5-Level EQ-5D
GAF: Global Assessment of Functioning
GDS-15: Geriatric Depression Scale-15
HAMD: Hamilton Depression Rating Scale
HAMD-17: Hamilton Rating Scale for Depression-17
HRQoL: Health-Related Quality of Life
HSUV: Health State Utility Value
HTA: Health Technology Assessment
ICD-10: International Classification of Diseases, 10th Revision
ID: Identifier
IQR: Interquartile Range
ISPOR: International Society for Pharmacoeconomics and Outcomes Research
MADRS: Montgomery-Åsberg Depression Rating Scale
MBCT-TS: Mindfulness-Based Cognitive Therapy with Support to Taper
MAOI: Monoamine Oxidase Inhibitor
MDD: Major Depressive Disorder
MDE: Major Depressive Episode
MeSH: Medical Subject Headings
MINI: Mini-International Neuropsychiatric Interview
NCT: National Clinical Trial
NICE: National Institute for Health and Care Excellence
NR: Not Reported
OL: Open Label
PDQ-D: Perceived Deficits Questionnaire–Depression
PPS: Painful Physical Symptoms
PRISMA: Preferred Reporting Items for Systematic Reviews and Meta-Analyses
QALY: Quality-Adjusted Life-Year
QIDS: Quick Inventory of Depressive Symptomatology
SCID: Structured Clinical Interview for DSM-IV
SD: Standard Deviation
SDS: Specialist Depression Services
RCT: Randomized Controlled Trial
SF-6D: Short Form six dimensions
SG: Standard Gamble
SNRI: Serotonin-Norepinephrine Reuptake Inhibitor
SSRI: Selective Serotonin Reuptake Inhibitor
TAU: Treatment as Usual
TC: Talking Control
TCA: Tricyclic Antidepressant
TESD: Treatment-Emergent Sexual Dysfunction
UK: United Kingdom
US: United States
VAS: Visual Analogue Scale
