# Supplementary material for: Health state utility values in major depressive disorder treated with pharmacological interventions: a systematic literature review
Source: Health Qual Life Outcomes. 2021 Mar 18;19:94. doi: 10.1186/s12955-021-01723-x (PMC7977292; doi:10.1186/s12955-021-01723-x)
Supplement: Supplementary file 1 — Additional file 1: PubMed literature search strategy. [file 12955_2021_1723_MOESM1_ESM.docx]

# ADDITIONAL FILE 1

1. PubMed Literature Search Strategy for Utility in Major Depressive Disorder (Search Conducted December 21, 2018)

| Term Group | Search No. | Search Terms | Hits |
| --- | --- | --- | --- |
| Population of interest | #1 | “Depressive Disorder, Major”[Majr] AND (“Drug Therapy”[Mesh] OR “Antidepressive Agents”[Mesh] OR “Antidepressive Agents”[Pharmacological Action] OR “Antipsychotic Agents”[Mesh] OR “Antipsychotic Agents”[Pharmacological Action]) | 7,399 |
|  | #2 | “Depressive Disorder, Major/drug therapy”[Majr] | 5,285 |
|  | #3 | #1 OR #2 | 7,933 |
| Utility | #4 | (((“health utility”[Text Word] OR “health utilities”[Text Word] OR “standard gamble”[Text Word] OR “time trade off”[Text Word] OR “time trade-off” OR “TTO”[Text Word] OR EuroQol* [Text Word] OR EQ5D* [Text Word] OR EQ 5D*[Text Word] OR EQ-5D*[Text Word] OR EuroQOL 5D*[Text Word] OR “HUI”[Text Word] OR “health utility index”[Text Word] OR “health utilities index”[Text Word] OR (health[Text Word] AND utilit*[Text Word] AND index[Text Word]) OR “SF-6D”[Text Word] OR sf6*[Text Word] OR sf 6*[Text Word] OR short form 6*[Text Word] OR shortform 6*[Text Word] OR “sf six”[Text Word] OR “sfsix”[Text Word] OR “shortform six”[Text Word] OR “short form six”[Text Word] OR “QALY”[Text Word] OR “Quality-Adjusted Life Years”[MeSH] OR “quality adjusted life year”[Text Word] OR “quality adjusted life years”[Text Word] OR “quality-adjusted life year”[Text Word] OR “quality adjusted life-year”[Text Word] OR “quality-adjusted life-year”[Text Word] OR “quality-adjusted life years”[Text Word] OR “quality adjusted life-years”[Text Word] OR “quality-adjusted life-years”[Text Word] OR “daly”[Text Word] OR “dalys”[Text Word] OR “disability adjusted life year”[Text Word] OR “disability adjusted life years”[Text Word] OR (utilit*[Text Word] AND score*[Text Word]) OR (utilit*[Text Word] AND weight*[Text Word])))) | 53,128 |
|  | #5 | #3 AND #4 | 106 |
| Exclusion terms | #6 | “Animals”[MeSH] NOT “Humans”[MeSH] | 4,529,722 |
|  | #7 | “Comment”[Publication Type] OR “Letter”[Publication Type] OR “Editorial”[Publication Type] OR “Case Reports”[Publication Type] OR “Clinical Trial, Phase I”[Publication Type] OR “case study”[Title] OR “case studies”[Title] OR “case report”[Title] OR “case reports”[Title] OR “case series”[Title] | 3,509,047 |
| All relevant studies | #8 | #5 NOT (#6 OR #7) | 106 |
|  | #9 | Filters: Publication date from 1998/01/01 | 106 |

HUI = Health Utilities Index; MeSH = Medical Subject Headings; SF-6D = Health Survey.
