# Supplementary material for: Health state utility values in major depressive disorder treated with pharmacological interventions: a systematic literature review
Source: Health Qual Life Outcomes. 2021 Mar 18;19:94. doi: 10.1186/s12955-021-01723-x (PMC7977292; doi:10.1186/s12955-021-01723-x)
Supplement: Supplementary file 2 — Additional file 2: Embase literature search strategy. [file 12955_2021_1723_MOESM2_ESM.docx]

# ADDITIONAL FILE 2

1. Embase Literature Search Strategy for Utility in Major Depressive Disorder (Search Conducted December 21, 2018)

| Term Group | Search No. | Search Terms | Hits |
| --- | --- | --- | --- |
| Population of interest | #1 | 'major depression'/exp/mj AND ('drug therapy'/exp OR 'antidepressant agent'/exp OR 'neuroleptic agent'/exp) | 12,094 |
|  | #2 | 'major depression'/exp/mj/dm_dt | 9,216 |
|  | #3 | #1 OR #2 | 12,463 |
| Utility | #4 | 'health utility':de,ab,ti OR 'health utilities':de,ab,ti OR 'standard gamble':de,ab,ti OR 'time trade off':de,ab,ti OR 'time trade-off':de,ab,ti OR 'tto':de,ab,ti OR euroqol*:de,ab,ti OR eq5d*:de,ab,ti OR ((eq NEXT/1 5d*):de,ab,ti) OR 'eq 5d*':de,ab,ti OR ((euroqol NEXT/1 5d*):de,ab,ti) OR 'hui':de,ab,ti OR 'health utility index':de,ab,ti OR 'health utilities index':de,ab,ti OR (health:de,ab,ti AND utilit*:de,ab,ti AND index:de,ab,ti) OR 'sf-6d':de,ab,ti OR sf6*:de,ab,ti OR 'sf 6':de,ab,ti OR 'short form 6':de,ab,ti OR 'shortform 6':de,ab,ti OR 'sf six':de,ab,ti OR 'sfsix':de,ab,ti OR 'shortform six':de,ab,ti OR 'short form six':de,ab,ti OR 'qaly':de,ab,ti OR 'quality adjusted life year'/exp OR 'quality adjusted life year':de,ab,ti OR 'quality adjusted life years':de,ab,ti OR 'quality-adjusted life year':de,ab,ti OR 'quality adjusted life-year':de,ab,ti OR 'quality-adjusted life-year':de,ab,ti OR 'quality-adjusted life years':de,ab,ti OR 'quality adjusted life-years':de,ab,ti OR 'quality-adjusted life-years':de,ab,ti OR 'daly':de,ab,ti OR 'dalys':de,ab,ti OR 'disability adjusted life year':de,ab,ti OR 'disability adjusted life years':de,ab,ti OR (utilit*:de,ab,ti AND score*:de,ab,ti) OR (utilit*:de,ab,ti AND weight*:de,ab,ti) | 94,748 |
|  | #5 | #3 AND #4 | 216 |
| Exclusion terms | #6 | 'animal'/exp NOT 'human'/exp | 5,162,063 |
|  | #7 | comment*:ti OR letter:it OR editorial:it OR 'case report'/exp OR 'phase 1 clinical trial'/exp OR 'case study':ti OR 'case studies':ti OR 'case report':ti OR 'case reports':ti OR 'case series':ti | 4,014,135 |
| All relevant studies | #8 | #5 NOT (#6 OR #7) | 212 |
|  | #9 | #8 AND [1998-2018]/py | 212 |

HUI = Health Utilities Index; SF-6D = Health Survey.
