# Supplementary material for: Health state utility values in major depressive disorder treated with pharmacological interventions: a systematic literature review
Source: Health Qual Life Outcomes. 2021 Mar 18;19:94. doi: 10.1186/s12955-021-01723-x (PMC7977292; doi:10.1186/s12955-021-01723-x)
Supplement: Supplementary file 3 — Additional file 3: Cochrane literature search strategy. [file 12955_2021_1723_MOESM3_ESM.docx]

# ADDITIONAL FILE 3

1. Cochrane Literature Search Strategy for Utility in Major Depressive Disorder (Search Conducted December 21, 2018)

| Term Group | Search No. | Search Terms | Hits |
| --- | --- | --- | --- |
| Population of interest | #1 | MeSH descriptor: [Depressive Disorder, Major] explode all trees | 4,044 |
|  | #2 | MeSH descriptor: [Drug Therapy] explode all trees | 132,574 |
|  | #3 | MeSH descriptor: [Antidepressive Agents] explode all trees | 5,360 |
|  | #4 | MeSH descriptor: [Antipsychotic Agents] explode all trees | 4,289 |
|  | #5 | #2 OR #3 OR #4 | 139,622 |
|  | #6 | #1 AND #5 | 1,725 |
|  | #7 | MeSH descriptor: [Depressive Disorder, Major] explode all trees and with qualifier(s): [drug therapy - DT] | 1,703 |
|  | #8 | #6 OR #7 | 2,101 |
| Utility | #9 | MeSH descriptor: [Quality-Adjusted Life Years] explode all trees | 1,050 |
|  | #10 | (“health utility” OR “health utilities” OR “standard gamble” OR “time trade off” OR “time trade-off” OR “TTO” OR EuroQol* OR EQ5D* OR EQ NEXT 5D* OR EQ-5D* OR EuroQOL NEXT 5D* OR “HUI” OR “health utility index” OR “health utilities index” OR (health AND utilit* AND index) OR “SF-6D” OR sf6* OR “sf 6” OR “short form 6” OR “shortform 6” OR “sf six” OR “sfsix” OR “shortform six” OR “short form six” OR “QALY” OR “quality adjusted life year” OR “quality adjusted life years” OR “quality-adjusted life year” OR “quality adjusted life-year” OR “quality-adjusted life-year” OR “quality-adjusted life years” OR “quality adjusted life-years” OR “quality-adjusted life-years” OR “daly” OR “dalys” OR “disability adjusted life year” OR “disability adjusted life years” OR (utilit* AND score*) OR (utilit* AND weight*)) | 14,572 |
|  | #11 | #9 OR #10 | 13,613 |
|  | #12 | #8 AND #11 | 56 |
| Exclusion terms | #13 | MeSH descriptor: [Animals] explode all trees | 16,570 |
|  | #14 | MeSH descriptor: [Humans] explode all trees | 8,532 |
|  | #15 | #13 NOT #14 | 8,038 |
|  | #16 | (“case study” OR “case studies” OR “case report” OR “case reports” OR “case series”):ti | 3,509,047 |
|  | #17 | (Comment OR Letter OR Editorial OR “Case Reports” OR “Clinical Trial, Phase I”):pt | 15,241 |
|  | #18 | #16 OR #17 | 16,303 |
| All relevant studies | #19 | #12 NOT (#15 OR #18) | 56 |
|  | #20 | Filters: Publication date from 1998/01/01 | 56 |

HUI = Health Utilities Index; MeSH = Medical Subject Headings; SF-6D = Health Survey.
