# Supplementary material for: Health state utility values in major depressive disorder treated with pharmacological interventions: a systematic literature review
Source: Health Qual Life Outcomes. 2021 Mar 18;19:94. doi: 10.1186/s12955-021-01723-x (PMC7977292; doi:10.1186/s12955-021-01723-x)
Supplement: Supplementary file 4 — Additional file 4: EconLit literature search strategy. [file 12955_2021_1723_MOESM4_ESM.docx]

# ADDITIONAL FILE 4

1. EconLit Literature Search Strategy for Utility in Major Depressive Disorder (Search Conducted December 21, 2018)

| Term Group | Search No. | Search Terms | Hits |
| --- | --- | --- | --- |
| Population of interest | S1 | depressive disorder, major | 19 |
|  | S2 | drug therapy | 88 |
|  | S3 | Antidepressive Agents | 0 |
|  | S4 | antipsychotic agents | 3 |
|  | S5 | S2 OR S3 OR S4 | 91 |
|  | S6 | S1 AND S5 | 0 |
|  | S7 | depressive disorder, major AND drug therapy | 0 |
|  | S8 | S6 OR S7 | 0 |
| Utility | S9 | “health utility” OR “health utilities” OR “standard gamble” OR “time trade off” OR “time trade-off” OR “TTO” OR EuroQol* OR EQ5D* OR EQ NEAR/1 5D* OR EQ-5D* OR EuroQOL NEAR/1 5D* OR “HUI” OR “health utility index” OR “health utilities index” OR (health AND utilit* AND index) OR “SF-6D” OR sf6* OR sf NEAR/1 6* OR short NEAR/1 form NEAR/1 6* OR shortform NEAR/1 6* OR “sf six” OR “sfsix” OR “shortform six” OR “short form six” OR “QALY” OR “quality adjusted life year” OR “quality adjusted life years” OR “quality-adjusted life year” OR “quality adjusted life-year” OR “quality-adjusted life-year” OR “quality-adjusted life years” OR “quality adjusted life-years” OR “quality-adjusted life-years” OR “daly” OR “dalys” OR “disability adjusted life year” OR “disability adjusted life years” OR (utilit* AND score*) OR (utilit* AND weight*) | 5,441 |
|  | S10 | S8 AND S9 | 0 |
| Exclusion terms | S11 | animals NOT humans | 2,184 |
|  | S12 | Comment* OR Letter OR Editorial OR “Case Reports” OR “phase I clinical trial” OR “case study” OR “case studies” OR “case report” OR “case reports” OR “case series” | 95,043 |
| All relevant studies | S13 | S11 OR S12 | 97,127 |
|  | S14 | S10 NOT S13 | 0 |

HUI = Health Utilities Index; SF-6D = SF-6D Health Survey.
