# Supplementary material for: Health state utility values in major depressive disorder treated with pharmacological interventions: a systematic literature review
Source: Health Qual Life Outcomes. 2021 Mar 18;19:94. doi: 10.1186/s12955-021-01723-x (PMC7977292; doi:10.1186/s12955-021-01723-x)
Supplement: Supplementary file 5 — Additional file 5: Study inclusion and exclusion criteria. [file 12955_2021_1723_MOESM5_ESM.docx]

# ADDITIONAL FILE 5

1. List of Criteria for the Inclusion and Exclusion of Studies During Level 1 and Level 2 Screening Process

| Criteria | Included | Excluded |
| --- | --- | --- |
| Population | - Adult patients (aged ≥ 18 years) - Patients with major depressive disorder | - Children (aged < 18 years) |
| Interventions and comparators | - Pharmacological treatments | - Studies of nonpharmacological treatments, e.g., exercise, Chinese medicine, any form of counselling therapy, electroconvulsive therapy |
| Outcomes | - Utility weights, by health state (e.g., EQ‑5D, SF-6D, or health utilities index data) - Utility decrements associated with adverse events | - Studies reporting quality-of-life data but not health-utility estimates |
| Study design | - Economic analyses reporting cost utility - Utility studies (including studies where utility weights were mapped from other instruments, such as disease-specific, patient-reported outcome measures) - Prospective studies reporting utility (e.g., observational studies, clinical trials) - Systematic reviews of economic analyses or utility studies^a^ | - Budget-impact analyses - Commentaries and letters (publication type) - Editorials - News articles - Nonsystematic reviews - Genetic or pathological studies - Case reports |
| Language | - English | - Articles not published in English |
| Date | - Articles published since January 1, 1998 - Conference abstracts published in 2016 or later | - Articles published before January 1, 1998 - Conference abstracts published before 2016 |

SF-6D = SF-6D Health Survey.

^a^ If it was unclear whether a study met any criterion during the level 1 screening process, the study was progressed to full-text screening to confirm its inclusion in the review.

^a^ Systematic reviews were included at level 1 screening, used for identification of primary studies, and then excluded at level 2 screening.
