# Supplementary material for: Health state utility values in major depressive disorder treated with pharmacological interventions: a systematic literature review
Source: Health Qual Life Outcomes. 2021 Mar 18;19:94. doi: 10.1186/s12955-021-01723-x (PMC7977292; doi:10.1186/s12955-021-01723-x)
Supplement: Supplementary file 6 — Additional file 6: Quality assessment of studies. [file 12955_2021_1723_MOESM6_ESM.docx]

# ADDITIONAL FILE 6

Table 1. Utility Weight Quality Assessment of Ang et al. (2009)

| Ang QQ, Wing YK, He Y, Sulaiman AH, Chiu NY, Shen YC, et al. Association between painful physical symptoms and clinical outcomes in East Asian patients with major depressive disorder: a 3-month prospective observational study. Int J Clin Pract. 2009;63(7):1041-9. | | |
| --- | --- | --- |
|  | Criteria/Question | Comment |
| **General Quality** | | |
|  | Study Sample Size | A total of 909 patients with MDD were enrolled. As this study was restricted to the psychiatric care setting only, it may have influenced and accounted for a lower proportion of patients with painful physical symptoms in this sample, and enrolled a disproportionately low ratio of inpatients to outpatients, which may have contributed to the overall low rate of hospitalizations during the study. |
|  | Respondent selection and recruitment | The study enrolled patients from 30 study sites across six East Asian countries and regions: China (Mainland), Hong Kong, Korea, Malaysia, Singapore, and Taiwan. Patients were recruited from 14 June 2006 to 15 February 2007, with individual patients being followed for a period of 3 months. No other details are provided. |
|  | Inclusion/exclusion criteria | The study included inpatients and outpatients, at least 18 years of age, who presented with a new or first episode of MDD, as defined by DSM-IV-TR or ICD-10 diagnostic criteria. All patients prepared to take antidepressant medication, were considered eligible for study entry. Additional inclusion criteria were CGI-S score ≥4 (moderate) at study entry, at least 2 months free of depression symptoms prior to onset of the present episode, and consent to the release of data. Participants were excluded if their current depressive episode had persisted for more than six continuous months, they had a previous or current diagnosis of schizophrenia, schizophreniform disorder, schizoaffective disorder, bipolar disorder or dementia, they were experiencing chronic treatment-resistant pain or pain of an inflammatory origin related to an identified medical condition, or they were simultaneously participating in another study that included treatment intervention and/or an investigational drug. |
|  | Response rates to instrument used | The overall completion rate was 79.0% (n = 718), with a significantly higher proportion of patient without painful physical symptoms completing the study (75.4% with symptoms, 82.9% without, p = 0.006). These data relate to all the measures used in the study, i.e., HAMD, CGI-S, VAS and EQ-5D – no separate response rate data is provided for EQ-5D. |
|  | Loss to follow-up | Patients were followed up for up to 3 months. 16.4% of patients with painful physical symptoms were lost to follow-up whereas only 10.3% of patients without symptoms were lost. No statistical analysis of the difference is provided. |
|  | Missing data | The method of dealing with missing data is not mentioned. |
|  | Any further problems with the study | The authors acknowledge several limitations of this study. Comparisons between antidepressant monotherapy groups were focused on patients who remained on their originally prescribed monotherapy for the entire study period, and thus the results may not provide an accurate representation of actual clinical practice, where medication switching is more common. Moreover, patients were included in the study based on their depression severity and as such, it is unknown whether the same findings would be observed if patients had been included based on their pain severity, which has been studied in various other cultural and regional settings. Similarly, the study did not ascertain how likely it would have been for patients to volunteer the presence of painful physical symptoms of their own accord. The fact that the study was restricted to the psychiatric care setting only, may have influenced and accounted for a lower proportion of patients with painful physical symptoms in this sample, and enrolled a disproportionately low ratio of inpatients to outpatients, which may have contributed to the overall low rate of hospitalizations during the study. The authors suggest future epidemiological studies which would also incorporate primary care and neurological settings and include a broader range of patients from the general population to improve understanding of the relationship between MDD and pain, particularly within East Asia. |
|  | Appropriateness of measure | The measures used in the study are valid to use in this group of patients. |
| **Relevance to HTA** | | |
|  | Do the population characteristics in the study match those modeled, and those described in the decision problem of the review? | Yes |
|  | What instrument is used to describe the health states? | EQ-VAS |
|  | From which population is the change in HRQoL undertaken? | Directly from the patient |
|  | From which population is the valuation of changes in the patients’ HRQoL undertaken? | Patient population |
|  | What technique is used to value the health states? | Visual analogue scale |

Table 2. Utility Weight Quality Assessment of Husain et al. (2017)

| Husain MI, Chaudhry IB, Husain N, Khoso AB, Rahman RR, Hamirani MM, et al. Minocycline as an adjunct for treatment-resistant depressive symptoms: A pilot randomised placebo-controlled trial. J Psychopharmacol. 2017;31(9):1166-75. | | |
| --- | --- | --- |
|  | Criteria/Question | Comment |
| **General Quality** | | |
|  | Study Sample Size | Initially, 87 potential study candidates were identified. However, 30 patients did not meet study inclusion criteria and 16 chose not to take part. Therefore, 41 participants were randomized, with 21 in the minocycline group and 20 in the PBO group. A total of 34 participants completed the trial. |
|  | Respondent selection and recruitment | The research clinician approached the clinical teams to inform them about the research study and the inclusion and exclusion criteria. If patients met the entry criteria, were clinically stable and the clinical team agreed that the patient could be a possible participant, they introduced the study to the patient. With the patient’s agreement, the research clinician then visited the patient to explain the research study verbally (in either Urdu or English) and to provide them with the participant information sheet (Urdu/English). The study was described to each potential participant with a witness (usually a caregiver) present. The patient had time to read and understand the patient information sheet (at least 24 hours). If they agreed to take part, a meeting (visit 1) was set up with the patient in order to obtain signed informed consent for the study and also signed consent for the research team to have access to their medical notes. Literate participants signed the consent forms but, if the participant could not write their name, they placed a thumbprint on the consent form which was countersigned by the witness. |
|  | Inclusion/exclusion criteria | Inclusion criteria were: (1) patients aged 18-65 years; (2) DSM-5 diagnosis of major depressive disorder; (3) capacity sufficient for consent to participate; (4) taking the current antidepressant medication for a minimum of 4 weeks (6 weeks for fluoxetine) prior to baseline; (5) the current episode of depression has failed to remit with at least two courses of antidepressant treatment (one of which is the current medication) at the adequate dose (according to BNF and Maudsley Prescribing Guidelines); relapse while taking an antidepressant is also considered a treatment failure; (6) able to take oral medication and (7) if female, willing to use adequate contraceptive precautions and to have monthly pregnancy tests. Exclusion criteria were: (1) relevant medical illness (renal, hepatic, cardiac, serious dermatological disorders such as exfoliative dermatitis, SLE); (2) prior history of intolerance to any of the tetracyclines; (3) concomitant penicillin therapy; (4) concomitant anticoagulant therapy; (5) presence of a seizure disorder; (6) currently taking valproic acid; (7) any change of psychotropic medications within the previous 4 weeks; (8) diagnosis of substance-use disorder (except nicotine or caffeine) or dependence within the last 3 months according to DSM-5 criteria; (9) pregnant or breastfeeding or (10) presence of primary psychotic disorder. |
|  | Response rates to instrument used | Response rate for EQ-5D is not reported. |
|  | Loss to follow-up | 2/21 (9.5%) of patients receiving minocycline were lost to follow-up. |
|  | Missing data | To deal with missing outcome data, the maximum likelihood approach was used: the main analysis used mixed effects models fit, using such models allow all available data to be included in the analysis, under the assumption that data is missing at random, that is, conditional on baseline predictors of missingness of outcomes being included in the model. To identify the latter, a binary indicator of missingness was generated for 12-week HAMD scores and predictors of missingness were sought using logistic regression and Fisher’s exact test. A criterion of p < 0.05 was used as with the small sample there is a risk of overfitting. There was a strong association between socio-economic status and missingness, p = 0.003, but no other predictors of missingness. Socio-economic status was therefore included in all the primary analysis models. |
|  | Any further problems with the study | The study findings should be interpreted in the light of certain limitations before minocycline can be recommended for routine clinical use. Firstly, as participants were followed up for a relatively short period of time, the authors were unable to comment on the long-term efficacy and safety of minocycline. Furthermore, as this was a pilot study with a small sample size, it requires replication in a larger sample. Future studies with larger sample sizes and longer follow-up periods are required to confirm the study findings. |
|  | Appropriateness of measure | The measures used in the study are valid to use in this group of patients. |
| **Relevance to HTA** | | |
|  | Do the population characteristics in the study match those modeled, and those described in the decision problem of the review? | Yes |
|  | What instrument is used to describe the health states? | EQ-VAS |
|  | From which population is the change in HRQoL undertaken? | Directly from the patient |
|  | From which population is the valuation of changes in the patients’ HRQoL undertaken? | Patient population. |
|  | What technique is used to value the health states? | Visual analogue scale. |

Table 3. Utility Weight Quality Assessment of Kuyken et al. (2015)

| Kuyken W, Hayes R, Barrett B, Byng R, Dalgleish T, Kessler D, et al. The effectiveness and cost-effectiveness of mindfulness-based cognitive therapy compared with maintenance antidepressant treatment in the prevention of depressive relapse/recurrence: results of a randomised controlled trial (the PREVENT study). Health Technol Assess. 2015;19(73):1-124. | | |
| --- | --- | --- |
|  | Criteria/Question | Comment |
| **General Quality** | | |
|  | Study Sample Size | Different relapse prevention interventions with different populations produce different absolute rates of depressive relapse/recurrence. Therefore, the sample size was based on estimated HRs for MBCT-TS compared with m-ADM rather than estimated absolute relapse/recurrence rates. The service users were canvassed and clinicians who concurred that a relative reduction in relapse/recurrence of 10% would be clinically important. We used the systematic review of MBCT compared with usual care for patients with recurrent depression that reported HRs of 0.28–0.47 for relapse/recurrence. Several conservative assumptions were applied. First, even though the pilot trial data suggest that the HR was improving in favor of MBCT as the length of follow-up increased, it was assumed a HR of 0.63 at 15 months to power the trial at 24-months’ follow-up. Second, even though attrition from MBCT trials to date is consistently <15%, it was assumed an attrition rate of 20% over the 24 months of follow-up. Finally, despite evidence to the contrary, it was assumed that there may be a small clustering effect (ICC = 0.01). This led to a total sample size of 420 across the two groups. For the secondary outcomes, meta-analyses of generic mindfulness approaches suggest medium effect sizes in terms of changes in depressive symptoms and the pilot trial suggested medium effect sizes for the secondary outcomes of residual depressive symptoms, psychiatric comorbidity and quality of life. The sample size estimate for our policy question enabled us to detect a medium between-groups effect size (standardized mean difference or Cohen’s d = 0.40) for the main secondary outcomes. |
|  | Respondent selection and recruitment | GP searches identified patients who had been prescribed ADM at a therapeutic dose in the last 3 months. GPs were then asked to screen this list to exclude any patients who they knew met the exclusion criteria. Letters were sent to the remaining patients enclosing an information pamphlet and reply form. Interested patients were telephoned to discuss the study and a short eligibility screening interview was conducted over the telephone. Information about the study and MBCT was available to help people to begin to make an informed decision about participation. This included the timings and locations of the MBCT groups. Most exclusions were identified at this stage. Patients who met the telephone screening criteria were invited to attend a face-to-face baseline interview. Consenting patients who met the PREVENT inclusion criteria joined the trial during the baseline assessment. Within a month of the start of the next MBCT-TS group a current GRID-HAMD score was obtained for each participant so that randomization could occur. Although most referrals were through GP surgeries, interested patients were also able to self-refer into the study. Several different strategies were applied to advertise the trial including placing posters in carefully targeted sites, developing a website, regional media coverage and leaflet dropping in local chemists. Patients were recruited in cohorts during recruitment ‘time slices’ that corresponded to the 6–8 weeks before the next MBCT-TS group was due to start. Baseline assessments were conducted as close as possible to the start of the MBCT-TS group as residual depressive symptoms are a powerful predictor of relapse/recurrence. On average, each researcher recruited six patients per month. |
|  | Inclusion/exclusion criteria | Participants were considered for inclusion if they: had a diagnosis of recurrent MDD in full or partial remission according to the DSM-IV l had had ≥3 previous major depressive episodes in which depression was the primary disorder and it was not secondary to substance abuse, bereavement or a general medical condition; were aged ≥18 years; were on a therapeutic dose of ADM in line with the BNF and NICE guidance; were open either to continue taking antidepressants for 2 years or to take part in a MBCT class and consider stopping their ADM. Participants were considered unsuitable for inclusion if they: were currently depressed, as assessed using the SCID for DSM-IV; had a comorbid diagnosis of current substance abuse (patients with previous substance abuse were eligible for inclusion as long as they were in sustained full remission); had organic brain damage; had current/past psychosis, including bipolar disorder; displayed persistent antisocial behavior; engaged in persistent self-injury that required clinical management/therapy; were undergoing formal concurrent psychotherapy. |
|  | Response rates to instrument used | At baseline, 413/424 (95%) patients provided valid EQ-5D-3L data. The rates were 347/424 (82%), 293/424 (69%), 324/424 (76%), 291/424 (69%), and 336/424 (70%) at 1-month, 9 months, 12 months, 18 months, and 24 months, respectively. |
|  | Loss to follow-up | In the MBCT-TS group, 17/212 (8.0%) were lost to follow-up at 1 month. The rates at 9 months, 12 months, 18 months, and 24 months were 23/212 (10.8%), 15/212 (7.1%), 20/212 (9.4%) and 10/212 (4.6%), respectively. The corresponding rates for patients in the m-ADM group were 20/212 (9.4%), 26/212 (12.3%), 18/212 (8.5%), 20/212 (9.4%), 10/212 (4.7%). |
|  | Missing data | At baseline, 2/424 (0.1%) patients had individual missing items within the valid cases. The rates were 0/424 (0%), 0/424 (0%), 1/424 (0.1%), 0/424 (0%), and 0/0 (70%) at 1-month, 9 months, 12 months, 18 months, and 24 months, respectively. Missing data were assumed missing at random and sensitivity analysis examined the effect of missing data using multiple imputations. Between-group inference for secondary outcome analyses was based on the complete case and imputed data sets are reported. Data entry and cleaning were overseen by the trial manager and research staff checked each outcome measure for missing data during every assessment and when possible collected missing items at this point. In cases in which ambiguous data were not clarified with the participant we operated a ‘score down policy’, meaning that if two items were checked the item with the lower rating was entered. When <10% of the total or subtotal items for one outcome were missing, the mean as an integer of the missing items subscale was imputed in place of the missing value. If > 10% of the total was missing then the whole questionnaire was recorded as missing and, if >10% of any one subtotal was missing, the whole of that subtotal was marked as missing. When substantive missing values arose, analyses were undertaken to assess their impact on the findings of the trial. Missing data were assumed to be ‘missing at random’,80 regression-based models were used to assess the relationship between covariates and outcome measure in completers and missing cases were substituted with a predicted outcome value. A sensitivity analysis (with and without imputed data) was undertaken to assess the potential impact of imputation on the trial findings. |
|  | Any further problems with the study | The recruitment strategy involved searching primary care databases and inviting patients who were currently taking m-ADM rather than recruiting patients who were discussing their options with their GP for preventing relapse/recurrence. The design included neither a usual care nor an attention control arm. The absence of an attention control arm means that any effects of MBCT-TS or m-ADM cannot be inferred to be specific to these treatments. The m-ADM arm included active monitoring of adherence by the research team and in this sense might best be represented as enhanced m-ADM. The pragmatic nature of the trial resulted in a proportion of patients in both arms not complying with the invitation to (dis)continue ADM. We undertook a per-protocol analysis to examine the impact on the primary outcome inference compared with ITT analysis. This is both a strength (pragmatism and generalizability) and a limitation (the ADM was not completely controlled). Finally, the sample consisted of a group at high risk of depressive relapse/recurrence currently taking ADM, who were open both to considering a group-based psychosocial treatment and to (dis)continuing their ADM. This is both a strength and limitation of the study. The findings of the PREVENT trial are therefore generalizable to only those individuals who are in equipoise about the type of preventative treatment that they choose, that is, m-ADM or switch to psychosocial intervention and reduce their ADM. |
|  | Appropriateness of measure | The measures used in the study are valid to use in this group of patients. |
| **Relevance to HTA** | | |
|  | Do the population characteristics in the study match those modeled, and those described in the decision problem of the review? | Yes |
|  | What instrument is used to describe the health states? | EQ-5D-3L. |
|  | From which population is the change in HRQoL undertaken? | Directly from the patient |
|  | From which population is the valuation of changes in the patients’ HRQoL undertaken? | Not reported by the study. |
|  | What technique is used to value the health states? | Not reported by the study. |

Table 4. Utility Weight Quality Assessment of Li et al. (2010)

| Li HC, Zhang MY, Wang G, Zhang HG, Zhang HY, Liu Y, et al. Association between painful physical symptoms and clinical outcomes in Chinese patients with major depressive disorder: a three-month observational study. Chin Med J (Engl). 2010;123(15):2063-9. | | |
| --- | --- | --- |
|  | Criteria/Question | Comment |
| **General Quality** | | |
|  | Study Sample Size | A total of 299 patients with MDD were enrolled. |
|  | Respondent selection and recruitment | This prospective, noninterventional, epidemiological observational study was designed to assess the frequency of painful physical symptoms among patients from East Asian countries and regions of China treated in naturalistic clinical practice settings for an acute episode of MDD. Recruitment was conducted from June 14, 2006 to February 15, 2007, with individual patients being followed for a period of 3 months. This sub-analysis presents data for all enrolled mainland Chinese patients who completed both baseline and endpoint visits. |
|  | Inclusion/exclusion criteria | Recruited patients included 108 (36.1%) inpatients and 191 (63.9%) outpatients of at least 18 years of age with either: a new or first episode of MDD as defined by the DSM-IV-TR diagnostic criteria; or a new or first depressive episode as defined by the ICD-10 diagnostic criteria, who were willing to take antidepressant medication. Additional inclusion criteria were a CGI-S score of ≥4 (moderate) at study entry; absence of depressive symptoms for ≥2 months prior to onset of the present episode, and formal consent to release data. Patients were not eligible for this study if they had current depressive episodes that had persisted for >6 continuous months; had a diagnosis of schizophrenia, schizophreniform disorder, schizoaffective disorder, bipolar disorder or dementia; were experiencing chronic treatment-resistant pain, or pain of an inflammatory origin related to an identified medical condition; or were participating in another study that included treatment intervention and/or an investigational drug. |
|  | Response rates to instrument used | Response rate for EQ-5D is not reported. |
|  | Loss to follow-up | The overall completion rate was 95% (n=284, 92.4% for patients with painful physical symptoms and 96.4% for patients without), and there were no significant differences between the groups regarding the reasons for discontinuation of treatment. |
|  | Missing data | This is not reported. |
|  | Any further problems with the study | A limitation of the present study is that this patient-based sample of individuals might not accurately reflect, despite its relatively large number, the characteristics of depression in the Chinese population. In this regard, the previously documented difficulty of Chinese patients to recognize and seek help for emotional complaints might have resulted patients with painful physical symptoms in seeking consultation in another medical specialty or simply remaining undiagnosed. This may also be a reasonable explanation for the present finding of a lower proportion of patients with painful physical symptoms in this sample compared with the overall population of East Asian MDD patients participating in the present study. Therefore, further studies that incorporate other clinical settings such as primary care, internal medicine or neurology, as well as population-based studies that investigate the prevalence of depression, the coexistence of painful physical symptoms, and their diagnosis and treatment are needed. |
|  | Appropriateness of measure | The measures used in the study are valid to use in this group of patients. |
| **Relevance to HTA** | | |
|  | Do the population characteristics in the study match those modeled, and those described in the decision problem of the review? | Yes |
|  | What instrument is used to describe the health states? | EQ-VAS |
|  | From which population is the change in HRQoL undertaken? | Directly from the patient |
|  | From which population is the valuation of changes in the patients’ HRQoL undertaken? | Patient population. |
|  | What technique is used to value the health states? | Visual analogue scale. |

Table 5. Utility Weight Quality Assessment of Montgomery et al. (2014)

| Montgomery SA, Nielsen RZ, Poulsen LH, Haggstrom L. A randomised, double-blind study in adults with major depressive disorder with an inadequate response to a single course of selective serotonin reuptake inhibitor or serotonin-noradrenaline reuptake inhibitor treatment switched to vortioxetine or agomelatine. Hum Psychopharmacol. 2014;29(5):470-82. | | |
| --- | --- | --- |
|  | Criteria/Question | Comment |
| **General Quality** | | |
|  | Study Sample Size | The all-patients-treated-set comprised 495 patients after the exclusion of six patients who did not take any study medication. The FAS comprised 493 patients after the exclusion of one patient from each treatment group with no valid postbaseline MADRS total score assessment. |
|  | Respondent selection and recruitment | This double-blind, randomized, flexible-dose, active comparator (agomelatine) study included 501 randomized patients recruited from 71 psychiatric inpatient and outpatient settings in 14 countries (Austria, Belgium, Bulgaria, Czech Republic, Estonia, Germany, Italy, Lithuania, Poland, Romania, Russia, Spain, Sweden and the UK) from January 2012 to December 2012. Patients were recruited via advertisements (in Austria, Germany, Estonia, Russia, Sweden, and the UK) or referrals from GPs. |
|  | Inclusion/exclusion criteria | Eligible patients were aged ≥18 and ≤75 years, with a primary diagnosis of a single episode or recurrent MDD according to the DDM-IV-TR and a current MDE of <12 months’ duration (confirmed using the MINI). Patients were required to have a MADRS total score ≥22 and item 1 (apparent sadness) score ≥3 at screening and baseline visits. Only patients with depressive symptoms considered nonresponsive or partially responsive to a single treatment course of an adequate dose (approved) and duration (≥6 weeks) were eligible for the study. In addition, patients had to want to change their current treatment because of an inadequate response and to be considered by the investigators to be candidates for a switch. Treatment resistance was excluded using both stage II and stage B criteria. Patients with a history of lack of response to agomelatine or previous exposure to vortioxetine were excluded. Patients were also excluded if they had any current axis I disorder other than GAD or SAD, as defined in the DSM-IV-TR and assessed using the MINI, or if they had a history of a manic or hypomanic episode, schizophrenia or any other psychotic disorder (including major depression with psychotic features), mental retardation, organic mental disorders or mental disorders because of a general medical condition, any substance abuse disorder within the previous 2 years, a history of a clinically significant neurological disorder, any neurodegenerative disorder or any axis II disorder that might compromise their participation in the study. Patients at serious risk of suicide, on the basis of the investigator’s clinical judgement, and those who had a score ≥5 on item 10 of the MADRS scale (suicidal thoughts) or had attempted suicide within <6 months were excluded, as were those receiving formal cognitive or behavioral therapy or systematic psychotherapy and pregnant or breastfeeding women. Patients were also excluded if they were taking disallowed concomitant medication, as well as the antibiotics rifampicin (broad inducer of CYP450 isoforms) and ciprofloxacin (potent CYP1A2 inhibitor contraindicated with agomelatine), although antiarrhythmics, antihypertensives and proton pump inhibitors (except cimetidine) were permitted. Episodic use of zolpidem, zopiclone or zaleplon for severe insomnia was allowed for a maximum of 2 days per week but not the night before a study visit. Patients were excluded if they had one or more clinical laboratory test values outside the reference range of potential risk to the patient’s safety or a serum ALT or AST >2xULN, a serum creatinine value >1.5xULN or a serum total bilirubin value >1.5xULN. This was due to hepatotoxicity concerns with agomelatine. |
|  | Response rates to instrument used | Response rate for EQ-5D is not reported. |
|  | Loss to follow-up | Only one patient was lost to follow-up during the study (in the vortioxetine group). |
|  | Missing data | This is not reported. |
|  | Any further problems with the study | The present study is a direct comparison of two effective antidepressants in inadequate responders to SSRI or SNRI treatment. The absence of PBO in the comparison makes it difficult to know whether the less effective treatment, agomelatine, was efficacious. The efficacy of vortioxetine by contrast is established because it was more effective than agomelatine under conditions of fair comparison, which is generally accepted as least as good if not better than PBO-controlled efficacy data. The influence of possible discontinuation symptoms on efficacy is another possible limitation. The attempt by investigators to minimize this by down titrating the dose of the previous antidepressant might well have minimized this problem. The failure to detect any difference in efficacy between the two treatments in the first 1 or 2 weeks when discontinuation symptoms are likely to be highest suggests that these symptoms did not influence the result. The study population of unsatisfactory response to one of a range of commonly used SSRIs or SNRIs, representing their approximate proportion of use in the general population, allows the results to be generalized to both SSRIs and SNRIs. However, the failure to include other classes of antidepressants means that the results may not be generalized confidently to other classes of antidepressants apart from the tricyclic antidepressants, which have a similar mechanism of action. The exclusion of comorbidity with other disorders (with the exception of GAD and SAD), which was necessary to allow confidence in addressing the specific question of the treatment of MDD, has had the effect of limiting the population studied to those with relatively pure depression, as in the PBO-controlled studies. Consequently, the results cannot necessarily be generalized to a population of MDD with high comorbidity. The significant advantage of vortioxetine on the HAM-A, however, suggests a potential advantage in those with comorbid anxiety, especially GAD and SAD. The limitation of excluding those at risk of suicide, those younger than 18 years, pregnant women and those who are excluded by regulatory restrictions, means that results cannot be confidently generalized to these groups. |
|  | Appropriateness of measure | The measures used in the study are valid to use in this group of patients. |
| **Relevance to HTA** | | |
|  | Do the population characteristics in the study match those modeled, and those described in the decision problem of the review? | Yes |
|  | What instrument is used to describe the health states? | EQ-VAS |
|  | From which population is the change in HRQoL undertaken? | Directly from the patient |
|  | From which population is the valuation of changes in the patients’ HRQoL undertaken? | Patient population. |
|  | What technique is used to value the health states? | Visual analogue scale. |

Table 6. Utility Weight Quality Assessment of Reed et al. (2009)

| Reed C, Monz BU, Perahia DG, Gandhi P, Bauer M, Dantchev N, et al. Quality of life outcomes among patients with depression after 6 months of starting treatment: results from FINDER. J Affect Disord. 2009;113(3):296-302. | | |
| --- | --- | --- |
|  | Criteria/Question | Comment |
| **General Quality** | | |
|  | Study Sample Size | 3,468 were included at baseline. |
|  | Respondent selection and recruitment | Patients were included in 6-month, European, prospective, observational study. |
|  | Inclusion/exclusion criteria | Primary care physicians or specialists (mostly psychiatrists) enrolled adult patients (≥18 years) presenting during the normal course of care with a clinical diagnosis of depression who were about to commence antidepressant treatment. |
|  | Response rates to instrument used | Response rate for EQ-5D is not reported. |
|  | Loss to follow-up | Of the 3,468 patients at baseline, 343 (9.9%) had no follow-up data, 271 (7.8%) had data at 3 months only, and 2854 (82.3%) had data at both 3 and 6 months or 6 months only. |
|  | Missing data | This is not reported. |
|  | Any further problems with the study | The FINDER study has several limitations. Firstly, important findings such as the association between HRQoL outcomes and between- and within-AD group switching need to be investigated further in more controlled settings. Secondly, HRQoL instruments partly measure concepts that are also contained in depression instruments. However, SF-36 and EQ-5D focus more on the impact on activities of daily living, social interactions and related aspects. Symptom severity and impact on everyday life are probably closely correlated, which may explain much of the parallel improvement in HRQoL and depression symptoms. Lastly, our observation period was limited to 6 months during which time patients did not approach general population values for the SF-36 MCS. Therefore, it was not possible to assess whether the time course to achieving mental HRQoL scores comparable to the general population is longer than 6 months or whether, even after treatment, participants remain impaired in this respect. |
|  | Appropriateness of measure | The measures used in the study are valid to use in this group of patients. |
| **Relevance to HTA** | | |
|  | Do the population characteristics in the study match those modeled, and those described in the decision problem of the review? | Yes |
|  | What instrument is used to describe the health states? | EQ-5D |
|  | From which population is the change in HRQoL undertaken? | Directly from the patient |
|  | From which population is the valuation of changes in the patients’ HRQoL undertaken? | Assumed to be general population |
|  | What technique is used to value the health states? | This is not reported. |

Table 7. Utility Weight Quality Assessment of Serfaty et al. (2009)

| Serfaty MA, Haworth D, Blanchard M, Buszewicz M, Murad S, King M. Clinical effectiveness of individual cognitive behavioral therapy for depressed older people in primary care: a randomized controlled trial. Arch Gen Psychiatry. 2009;66(12):1332-40. | | |
| --- | --- | --- |
|  | Criteria/Question | Comment |
| **General Quality** | | |
|  | Study Sample Size | A total of 376 people were referred for assessment. 45% (n=170) were self-referrals, 27% (n=101) GP referrals, and 28% (n=105) recruited from GP database searches. Of these, 54% (n=204) met the inclusion criteria; 41% (n=83) were self-referrals, 31% (n=63) were GP referrals, and 28% (n=58) were referrals from database searches. |
|  | Respondent selection and recruitment | This was a single-blind, randomized, controlled trial in which the researchers were blind to group allocation until all data entry had been completed. The study took place between April 2004 and September 2007. 47 general practices were selected through the North Central Thames General Practice Research Network, each with a mean (SD) of 6.8 (3.0) practitioners per practice and covering an area in North London (boroughs of Camden, Islington, Barnet, Enfield, and Haringey). The population of people >65 years in this area is just greater than 140 000. People aged ≥65 years or older were recruited by methods developed from previous feasibility work, namely self-referral, general practice referral (either from a direct approach by researchers or via the GP), and by database searches. The 15-item Geriatric Depression Scale, a self-rated questionnaire for depression in the elderly was used for screening. Participants who scored 5 or higher were offered a further interview to see whether they satisfied the entry criteria. |
|  | Inclusion/exclusion criteria | The inclusion criteria were (1) a primary diagnosis of depressive disorder made by the researcher who administered the Geriatric Mental State and History and Etiology Schedule using the computerized diagnostic program AGECAT, which has been validated in the community and used in international comparisons of depression; (2) a score of ≥14 on the BDI-II to include people with less severe mixed anxiety and depression, who are frequently seen in primary care and who may respond to CBT; (3) sufficient command of English to use CBT techniques; and (4) if taking an antidepressant, a stable dose of medication for at least 8 weeks prior to randomization. The exclusion criteria were (1) intense suicidal intent requiring inpatient admission; (2) a history suggestive of alcohol misuse or drug dependence; (3) a history of bipolar affective disorder; (4) the presence of hallucinations or delusions; (5) cognitive deficits, as judged by a score of <24 on the Mini-Mental State Examination; (6) having received CBT in the last year; and (7) having received electroconvulsive therapy within the previous 6 months, because of possible residual effects on cognition. |
|  | Response rates to instrument used | All patients had Euroqol data at baseline. At post-intervention assessment 1, 61/70 (87.1%) patients treated with CBT completed the questionnaire, whereas 57/67 (85.1%) and 55/67 (82.1%) patients treated with talking control and treatment as usual had data, respectively. At post-intervention assessment 2 the corresponding rates were 56/70 (80.0%), 53/67 (79.1%), and 50/67 (74.6%). |
|  | Loss to follow-up | Lost to follow-up data is not reported. |
|  | Missing data | This is not reported. |
|  | Any further problems with the study | Potential biases (blindness, therapist’s factors, intervention issues, antidepressant use) need to be considered when evaluating the study. It was difficult for researchers to remain masked to group allocation. However, participants completed self-rating assessments of mood; therefore, the lack of blindness should not have affected our primary outcome to any great extent. Therapist skills and their allegiance to treatment influence outcome. Multiple therapists delivering the intervention may be used to control for both known and unknown therapist factors, with some adjustments being made in sample size for a therapist cluster effect. An alternative approach was to standardize the interventions (manualized CBT and a TC) and control for nonspecific therapist skills (e.g., empathy and warmth. |
|  | Appropriateness of measure | The measures used in the study are valid to use in this group of patients. |
| **Relevance to HTA** | | |
|  | Do the population characteristics in the study match those modeled, and those described in the decision problem of the review? | Yes |
|  | What instrument is used to describe the health states? | EQ-5D |
|  | From which population is the change in HRQoL undertaken? | Directly from the patient |
|  | From which population is the valuation of changes in the patients’ HRQoL undertaken? | Not reported by the study. |
|  | What technique is used to value the health states? | Not reported by the study. |

Table 8. Utility Weight Quality Assessment of Papakostas et al. (2018)

| Papakostas GI, Nielsen RZ, Dragheim M, Tonnoir B. Efficacy and tolerability of vortioxetine versus agomelatine, categorized by previous treatment, in patients with major depressive disorder switched after an inadequate response. J Psychiatr Res. 2018;101:72-9. | | |
| --- | --- | --- |
|  | Criteria/Question | Comment |
| **General Quality** | | |
|  | Study Sample Size | The all-patients-treated-set comprised 493 patients after excluding two patients not previously treated with an SSRI/SNRI. The FAS comprised 491 patients after excluding one patient from each treatment group who had no valid postbaseline MADRS total score assessment. |
|  | Respondent selection and recruitment | This was a DB, randomized, 12-week comparator study. Before inclusion in this study, eligible patients had to have been considered by the investigator to be non- or partially responsive to no more than 1 adequate course (≥6 weeks) of an approved dose of SSRI/SNRI monotherapy (citalopram, escitalopram, paroxetine, sertraline, duloxetine, or venlafaxine) and were candidates for a switch. In addition, the patient wished to change antidepressant because of inadequate response. Investigators were advised to down-titrate the SSRI/SNRI to the lowest effective antidepressant dose in the week before the baseline visit and randomization to agomelatine or vortioxetine. Patients were switched directly and randomized (1:1) to vortioxetine (10–20 mg/day) or agomelatine (25–50 mg/day). |
|  | Inclusion/exclusion criteria | Eligible patients were aged ≥18 and ≤75 years, with a primary diagnosis of a single major depressive episode or recurrent MDD (DSM IV-TR criteria), a current MDE of <12 months' duration, a MADRS total score of ≥22, and an item 1 (apparent sadness) score of ≥3 at screening and baseline visits. |
|  | Response rates to instrument used | **MMRM:**  Vortioxetine (prior SSRIs):  Week 8: 164/190 (86.3%)  Week 12: 151/190 (79.5%)  Vortioxetine (prior SNRIs):  Week 8: 56/62 (9.0%)  Week 12: 49/62 (7.9%)  Agomelatine (prior SSRIs):  Week 8: 149/188 (79.3%)  Week 12: 141/188 (75.0%)  Agomelatine (prior SNRIs):  Week 8: 40/53 (75.5%)  Week 12: 37/53 (69.8%)  **ANCOVA (LOCF)**  Vortioxetine (prior SSRIs):  Week 8: 181/190 (95.3%)  Week 12: 181/190 (95.3%)  Vortioxetine (prior SNRIs):  Week 8: 61/62 (98.4%)  Week 12: 61/62 (98.4%)  Agomelatine (prior SSRIs):  Week 8: 183/188 (97.3%)  Week 12: 183/188 (97.3%)  Agomelatine (prior SNRIs):  Week 8: 49/53 (92.5%)  Week 12: 49/53 (92.5%) |
|  | Loss to follow-up | None of the patients who had received prior SSRIs were lost to follow-up. One patient who received vortioxetine and received prior SNRIs was lost to follow-up (1/62, 1.6%). |
|  | Missing data | This is not reported. |
|  | Any further problems with the study | When interpreting the present findings, clinicians should also keep in mind several potential limitations of these subgroup analyses which were not powered to show differences for the individual subgroups. First, it should be noted that patients were not randomized or stratified by prior treatment group (SSRI or SNRI), although a broad array of measures did not appear to differ significantly between the two treatment groups at baseline. Second, although the current sample size is sufficient to conduct a broad analysis based on antidepressant class, much larger sample sizes would be required to be able to make more definitive statements based on the six individual agents (i.e., citalopram, escitalopram, paroxetine, sertraline, duloxetine, and venlafaxine), though we saw a trend toward improvement with vortioxetine independent of the previous treatment. In addition, not all antidepressants in the SSRI or SNRI category are represented in the current sample (i.e., fluoxetine, fluvoxamine, desvenlafaxine, milnacipran, and levomilnacipran); however, it is unclear whether the results would have been different if patients who were partial- or non-responders to these agents were included as well. Finally, clinical trials employ several exclusion criteria (e.g., high levels of suicidal ideation, mild forms of depression) which, to varying degrees, may affect the generalizability of the current findings to excluded populations. |
|  | Appropriateness of measure | The measures used in the study are valid to use in this group of patients. |
| **Relevance to HTA** | | |
|  | Do the population characteristics in the study match those modeled, and those described in the decision problem of the review? | Yes |
|  | What instrument is used to describe the health states? | EQ-VAS |
|  | From which population is the change in HRQoL undertaken? | Directly from the patient |
|  | From which population is the valuation of changes in the patients’ HRQoL undertaken? | Patient population. |
|  | What technique is used to value the health states? | Visual analogue scale. |

Table 9. Utility Weight Quality Assessment of Saragoussi et al. (2018)

| Saragoussi D, Christensen MC, Hammer-Helmich L, Rive B, Touya M, Haro JM. Long-term follow-up on health-related quality of life in major depressive disorder: a 2-year European cohort study. Neuropsychiatr Dis Treat. 2018;14:1339-50. | | |
| --- | --- | --- |
|  | Criteria/Question | Comment |
| **General Quality** | | |
|  | Study Sample Size | A total of 1,159 patients completed the baseline visit and at least one follow-up visit without any violation of inclusion or exclusion criteria and were therefore included in the analysis. |
|  | Respondent selection and recruitment | PERFORM was a 2-year multicenter, prospective, noninterventional cohort study in outpatients with MDD enrolled by either a GP or a psychiatrist at 194 sites in France, Germany, Spain, Sweden, and the UK (NCT01427439). |
|  | Inclusion/exclusion criteria | Eligible patients were aged 18–65 years, had a current diagnosis of MDD according to the DSM-IV-TR criteria; confirmed by the Mini-International Psychiatric Interview questionnaire (depression module), and were either initiating antidepressant monotherapy or undergoing their first switch of antidepressant. The choice of antidepressant prescribed was determined by the treating physician and was independent of study participation. Patients receiving antidepressant combination therapy at the time of the initial consultation and patients with schizophrenia or other non-affective psychosis, bipolar disorder, substance dependence, mood disorders due to a general medical condition or substances, or dementia or other neurodegenerative diseases that might significantly impact cognitive functioning were excluded from study entry. Pregnant women and women ≤6 months postpartum were also excluded. |
|  | Response rates to instrument used | A total of 862 patients (74.4% of total) completed the full 2 years of follow-up. At month 2, 201/259 (77.6%) provided data for EQ-5D. |
|  | Loss to follow-up | This is not reported. |
|  | Missing data | Missing data were not replaced in any of the analyses (i.e., all data included in the analyses were observed cases). |
|  | Any further problems with the study | Analysis of the association between cognitive symptoms and HRQoL assessed using the EQ-5D specifically was limited by the fact that the questionnaire was only used in the UK, thereby reducing the statistical power of the analysis. This may at least in part explain why the observed association between severity of cognitive symptoms and EQ-5D utility index score at baseline was not seen at subsequent time points as was observed for the SF-12 MCS and PCS, although this could also be due to the greater association of EQ-5D utility index score with other factors, such as depression severity. Further, the study only recruited outpatients who were initiating antidepressant monotherapy or switching antidepressant monotherapy for the first time; this means that the study results cannot be generalized to the entire MDD population in Europe, particularly those at a later disease stage or those receiving psychotherapy only. |
|  | Appropriateness of measure | The measures used in the study are valid to use in this group of patients. |
| **Relevance to HTA** | | |
|  | Do the population characteristics in the study match those modeled, and those described in the decision problem of the review? | Yes |
|  | What instrument is used to describe the health states? | EQ-5D |
|  | From which population is the change in HRQoL undertaken? | Directly from the patient |
|  | From which population is the valuation of changes in the patients’ HRQoL undertaken? | UK general population. |
|  | What technique is used to value the health states? | Time trade-off when using the UK tariff. |

Table 10. Utility Weight Quality Assessment of Morriss et al. (2016)

| Morriss R, Garland A, Nixon N, Guo B, James M, Kaylor-Hughes C, et al. Efficacy and cost-effectiveness of a specialist depression service versus usual specialist mental health care to manage persistent depression: a randomised controlled trial. Lancet Psychiatry. 2016;3(9):821-31. | | |
| --- | --- | --- |
|  | Criteria/Question | Comment |
| **General Quality** | | |
|  | Study Sample Size | 307 patients were referred to the study and assessed for eligibility. 228 patients (74%) completed a baseline interview, with non-progression at this stage attributable to various factors. 41 patients (13%) of initial referrals were either screened out or withdrew after the baseline interview, so that 187 patients (61%) were randomly assigned to a treatment group. |
|  | Respondent selection and recruitment | All participants were referred to the study by a mental health professional working in one of the three participating specialist mental health organizations. This was a multicenter, single-blind, patient-level, parallel group, randomized controlled study in secondary care psychiatry settings within three NHS trusts in England (Derbyshire Healthcare NHS Foundation Trust, Nottinghamshire Healthcare NHS Foundation Trust, and Cambridgeshire and Peterborough NHS Foundation Trust). 307 patients were referred to the study and assessed for eligibility between Dec 21, 2009, and Oct 31, 2012, as part of the NIHR CLAHRC long-term follow-up of patients with persistent depression study. 228 patients (74%) completed a baseline interview, with non-progression at this stage attributable to various factors. 41 patients (13%) of initial referrals were either screened out or withdrew after the baseline interview, so that 187 patients (61%) were randomly assigned to a treatment group. 21 patients (11%) were from the Derby site, 137 (73%) from the Nottingham site, and 29 (16%) were from the Cambridge site. One participant was randomly assigned at the Derby site but received treatment and was followed up by the Cambridge site because of the greater convenience of this arrangement to the patient; for the purposes of analysis, this patient is included as per randomization in the Derby group of the study. |
|  | Inclusion/exclusion criteria | Patients with persistent moderate or severe depression were recruited. The definition of persistent depression was deliberately pragmatic, encouraging the referral of patients who had not responded to secondary mental health care for at least 6 months, and in whom their main current problem was depression. Inclusion criteria were that the patient was thought by the referrer to have primary unipolar depression (i.e., not caused by another psychiatric disorder); aged older than 18 years; able and willing to give oral and written informed consent to participate in the study; must have been offered or received direct and continuous care from one or more health professionals in the preceding 6 months and currently be under the care of a secondary care mental health team; had a structured clinical interview for DSM-IV (SCID) diagnosis of major depressive disorder with a current major depressive episode; met 5/9 NICE criteria for symptoms of moderate depression; had a HDRS17 ≥16; and had a GAF score of ≤60, to ensure that the patient had a current moderate or severe major depressive episode. Patients were excluded if they were in receipt of emergency care for suicide risk, at risk of severe neglect, or a homicide risk, but patients were not excluded because of such risk provided the risk was adequately contained in their current care setting and the primary medical responsibility for care was with the referral team. They were also excluded if they did not speak fluent English; were pregnant; or had unipolar depression secondary to a primary psychiatric or medical disorder, except when bipolar disorder was identified by the research team after referral as unipolar depression because an SDS would be expected to manage bipolar depression in clinical practice. |
|  | Response rates to instrument used | For both groups, 144 patients (77%) were assessed at 6 months’ follow-up, 134 patients (72%) at 12 months, and 110 (59%) patients were assessed at 18 months’ follow-up. Unmasking events of outcome assessor were reported during follow-up in 71 patients (38%). Response rates are not reported. |
|  | Loss to follow-up | Between treatment assignment and 6-month follow-up, 28/94 (29.8%) patients who received treatment as usual were lost to follow-up and 15/93 (16.1%) patients in the specialist depression services group were lost. 66 patients received treatment as usual at 6-months and 7/66 (10.6%) were lost at 12-months follow-up. The corresponding number for patient receiving specialist depression services was 4/78 (5.1%). The numbers of patients lost to follow-up at 18 months were 11/59 (18.6%) and 13/75 (17.3%), respectively. |
|  | Missing data | Markov chain Monte Carlo multiple imputation was used to impute missing data with REALCOM software under a missing-at-random assumption after exploration of the effect of the observed data on missing values. To assess the robustness of the results and sensitivity to missing values, a multilevel modeling was also run with only observed scores. A similar multilevel modeling was done to analyze GAF scores and all secondary outcome variables. A covariate adjustment analysis was done by including site as a covariate in multilevel modeling. A sensitivity analysis was run for only observed data (excluding missing data) for the primary outcomes and secondary depression outcomes to check how the results of the main analyses would be affected by exclusion of these variables and also by any participants without 6, 12, or 18 month outcomes. |
|  | Any further problems with the study | A larger sample size was recruited than stated in the published protocol because the number of patients in follow-up at 12 months was lower than anticipated. Nevertheless, the study might have been underpowered to detect statistical improvement in symptoms and function at 12 months’ follow-up. Patients were more severely ill and had been ill for longer than was expected, thus requiring longer durations of specialist depression services treatment and a graduated transfer to aftercare with clear recommendations for ongoing care that extended after the 12 months’ primary outcome endpoint. As a result, and in line with NICE recommendations for research into service interventions for depression, the outcomes were reported at 18 months’ and 12 months’ follow-up. Many patients were lost during follow-up in this study. An important limitation of both the clinical and economic outcomes is therefore the loss of participants to follow-up between 12 months and 18 months, which reduces the certainty of the results. However, in this study, imputed and original clinical outcomes were very similar. Another important limitation of the study was that full masking proved impossible to achieve. |
|  | Appropriateness of measure | The measures used in the study are valid to use in this group of patients. |
| **Relevance to HTA** | | |
|  | Do the population characteristics in the study match those modeled, and those described in the decision problem of the review? | Yes |
|  | What instrument is used to describe the health states? | EQ-5D |
|  | From which population is the change in HRQoL undertaken? | Directly from the patient |
|  | From which population is the valuation of changes in the patients’ HRQoL undertaken? | UK general population. |
|  | What technique is used to value the health states? | Time trade-off when using the UK tariff. |

Table 11. Utility Weight Quality Assessment of Revicki et al. (1998)

| Revicki DA, Wood M. Patient-assigned health state utilities for depression-related outcomes: differences by depression severity and antidepressant medications. J Affect Disord. 1998;48(1):25-36. | | |
| --- | --- | --- |
|  | Criteria/Question | Comment |
| **General Quality** | | |
|  | Study Sample Size | The total of 70 patients were enrolled into the study. |
|  | Respondent selection and recruitment | Patients were recruited from two centers, 40 patients from a university family practice in Toronto, Canada, and 30 from a community-based primary care practice in San Diego, US. |
|  | Inclusion/exclusion criteria | Patients were aged between 18-65 years with a DSM-III-R diagnosis of MDD, based on a clinician interview using the SCI for DSM Disorders. Patients with dysthymia were included if they also met DSM-III-R criteria for major depression disorder in the previous 6 months. Patients completed at least 8 weeks of antidepressant treatment or had completed an antidepressant treatment with the last 2 months. Patients were excluded if they had a concurrent DSM-III-R diagnosis of mood disorders other than MDD, organic brain disease or known malignancy or had received ECT during the current or most recent depression episode. |
|  | Response rates to instrument used | Response rate to standard gamble interviews was not reported |
|  | Loss to follow-up | Loss to follow-up was not reported. |
|  | Missing data | Twelve patients had missing or incomplete health utility data, nine patients had missing, or incomplete health status data and one patient had missing or incomplete clinical data. |
|  | Any further problems with the study | The study is 22 years old, so the treatments listed in the study may not be standard of care any longer. The researchers acknowledged that the sample size is relatively small, which may limit the ability of the study to identify small and meaningful differences in the utilities. The population was a convenience sample rather than a randomized trial, so may not be representative of the wider population. Finally, the study did not account for comorbidities or other chronic conditions which may impact on the utility of patients. |
|  | Appropriateness of measure | The measures were valid for the population. |
| **Relevance to HTA** | | |
|  | Do the population characteristics in the study match those modeled, and those described in the decision problem of the review? | Yes |
|  | What instrument is used to describe the health states? | The health states were constructed based on reviews of the psychiatric literature and the consultation of three psychiatrists with experience in the diagnosis and treatment of depression. |
|  | From which population is the change in HRQoL undertaken? | Directly from the patients. |
|  | From which population is the valuation of changes in the patients’ HRQoL undertaken? | Not reported by the study. |
|  | What technique is used to value the health states? | Standard gamble |

Table 12. Utility Weight Quality Assessment of Garcia-Cebrian et al. (2008)

| Garcia-Cebrian A, Bauer M, Montejo AL, Dantchev N, Demyttenaere K, Gandhi P, et al. Factors influencing depression endpoints research (FINDER): study design and population characteristics. Eur Psychiatry. 2008;23(1):57-65. | | |
| --- | --- | --- |
|  | Criteria/Question | Comment |
| **General Quality** | | |
|  | Study Sample Size | 3,515 patients were enrolled in the study, with 3,468 patients eligible for inclusion. |
|  | Respondent selection and recruitment | Patients were recruited from centers in 12 European countries: Austria, Belgium, France, Germany, Ireland, Italy, Netherlands, Norway, Portugal, Sweden, Switzerland, and the UK. Patients were enrolled between May 2004 and September 2005. Data were collected at baseline (routine visit where patient agreed to partake in study), and then 3- and 6-months postbaseline (again during routine clinical visits). Austria n = 163, Belgium n = 239, France n = 606, Germany n = 649, Ireland n = 68, Italy n = 513, the Netherlands n = 196, Norway n = 40, Portugal n = 48, Sweden n = 199, Switzerland n = 139, and the UK n = 608. |
|  | Inclusion/exclusion criteria | Patients were eligible for inclusion if they presented within the normal course of care and (1) were clinically diagnosed by their physician as suffering from depression, (2) were about to start antidepressant pharmacological treatment for either a first or subsequent episode of depression (the index episode), (3) were aged at least 18 years, and (4) were not simultaneously participating in another study that involved an investigational drug or procedure. |
|  | Response rates to instrument used | Only baseline responses were reported on. |
|  | Loss to follow-up | Loss to follow-up is not reported. |
|  | Missing data | 1.3% of enrolled patients were not eligible for enrollment. For analysis, all items within each HADS subscale had to be answered otherwise the subscale was set to missing. Patients were excluded from the analysis if one or more entry criteria were violated or from individual analyses based on missing, implausible (according to pre-defined ranges) or uninterpretable data. The exclusion of patients’ data from analysis was based on study entry criteria at the first observation, not on whether or not antidepressant medication was taken during the 6 month follow-up period. |
|  | Any further problems with the study | The researchers identified several limitations to the study: (1) the sample was limited to patients initiating antidepressant treatment, thus excluding those with unrecognized depression, those untreated and those exclusively receiving a nonpharmacological treatment; (2) data collected retrospectively for the previous 2 years before study entry may be affected by recall bias; (3) the prespecified lists of psychiatric, physical and functional conditions may not be comprehensive enough to capture all comorbid conditions present; (4) the prespecified list may have also prompted physicians to record certain disorders that may not withstand further diagnostic confirmation; (5) HRQoL instruments used in the study (e.g., SF-36 and EQ-5D) partly measure concepts that are also contained in depression instruments. As an observational study, there is more opportunity for bias to affect the results compared with an RCT. |
|  | Appropriateness of measure | The measures were valid for the population. |
| **Relevance to HTA** | | |
|  | Do the population characteristics in the study match those modeled, and those described in the decision problem of the review? | Yes |
|  | What instrument is used to describe the health states? | EQ-5D and SF-36 |
|  | From which population is the change in HRQoL undertaken? | Directly from the patients. |
|  | From which population is the valuation of changes in the patients’ HRQoL undertaken? | Not reported by the study. |
|  | What technique is used to value the health states? | Not reported by the study. |

Table 13. Utility Weight Quality Assessment of Sapin et al. (2004)

| Sapin C, Fantino B, Nowicki ML, Kind P. Usefulness of EQ-5D in assessing health status in primary care patients with major depressive disorder. Health Qual Life Outcomes. 2004;2:20. | | |
| --- | --- | --- |
|  | Criteria/Question | Comment |
| **General Quality** | | |
|  | Study sample size | The study sample size was 250 patients. |
|  | Respondent selection and recruitment | 95 French primary care practitioners recruited 250 patients with a DSM-IV diagnosis of MDD for inclusion in an eight-week follow-up cohort. |
|  | Inclusion/exclusion criteria | Patients were aged over 18 years old and had consulted a GP for a new episode of MDD according to the DSM-IV and had not been treated with any antidepressant before inclusion. Patients with symptoms that suggested non-MDD illness were excluded. |
|  | Response rates to instrument used | Response rates were not reported for each instrument. |
|  | Loss to follow-up | Among the 250 included patients, 24 were lost to follow-up (9.6%). |
|  | Missing data | The patient baseline characteristics, patient gender was missing for one patient, professional status was missing for four patients and place of residence was missing for 26 patients. |
|  | Any further problems with the study | The researchers acknowledged that there were several limitations to the study. First, the study does not consider the antidepressant prescribed or their side effects, which may influence patients' ratings. Second, the concomitant impact of depression and chronic medical conditions could not be examined in this sample. It is likely that the health state utilities of patients with depression, in addition to a chronic medical disease would be significantly reduced. Lastly, a limitation of the analysis presented in this study relates to the source of the utility weights used to compute the EQ-5D. Given that this was a national study conducted in France it may have been better to use social preference values based on the French population. |
|  | Appropriateness of measure | The measures were valid for the population. |
| **Relevance to HTA** | | |
|  | Do the population characteristics in the study match those modeled, and those described in the decision problem of the review? | Yes |
|  | What instrument is used to describe the health states? | SF-36 and EQ-5D |
|  | From which population is the change in HRQoL undertaken? | Directly from the patients. |
|  | From which population is the valuation of changes in the patients’ HRQoL undertaken? | UK population tariff |
|  | What technique is used to value the health states? | Time trade-off |

Table 14. Utility Weight Quality Assessment of Lee et al. (2009a)

| Lee P, Zhang M, Hong JP, Chua HC, Chen KP, Tang SW, et al. Frequency of painful physical symptoms with major depressive disorder in Asia: relationship with disease severity and quality of life. J Clin Psychiatry. 2009;70(1):83-91. | | |
| --- | --- | --- |
|  | Criteria/Question | Comment |
| **General Quality** | | |
|  | Study Sample Size | The study sample size was 909 enrolled patients. |
|  | Respondent selection and recruitment | Patients were enrolled from 40 study sites across six Asian countries and regions (China, Hong Kong, Korea, Malaysia, Singapore, and Taiwan), from the psychiatric care setting between June 14, 2006 and February 15, 2007. Patients were followed for a period of 3 months. |
|  | Inclusion/exclusion criteria | Included patients were inpatients or outpatients, aged at least 18 years old, who presented with a new or first episode of MDD, as defined by DSM-IV-TR or ICD-10. Patients had to be prepared to take antidepressant medication. Additionally, patients had to have a CGI-S score ≥4 at study entry, at least 2 months free of depressive symptoms prior to onset of new/first episode and to consent to release of data. Patients were excluded if their current episode of depression had been persistent for more than 6 months, a previously diagnosed other mental health illness, if they were experiencing chronic, treatment-resistant pain or if they were simultaneously participating in another study that included treatment intervention or investigational drug. |
|  | Response rates to instrument used | Only baseline data was reported by the study. |
|  | Loss to follow-up | Loss to follow-up was not reported. |
|  | Missing data | Missing data was not reported by the study. |
|  | Any further problems with the study | The researchers identified several limitations of the study. The researchers could not be certain that the Asian population with MDD was comparable with other populations. The researchers could have collected information on comorbidity anxiety disorders, as there is evidence that a large proportion of anxiety patients also experience painful physical symptoms. Additionally, as an observational study the researchers should have acknowledged that the observational studies are inherently more likely to experience bias in the results compared to an RCT. |
|  | Appropriateness of measure | The measures were valid for the population. |
| **Relevance to HTA** | | |
|  | Do the population characteristics in the study match those modeled, and those described in the decision problem of the review? | Yes |
|  | What instrument is used to describe the health states? | EQ-5D |
|  | From which population is the change in HRQoL undertaken? | Directly from the patients. |
|  | From which population is the valuation of changes in the patients’ HRQoL undertaken? | Not reported by the study. |
|  | What technique is used to value the health states? | Not reported by the study. |

Table 15. Utility Weight Quality Assessment of Lee et al. (2009b)

| Lee MS, Yum SY, Hong JP, Yoon SC, Noh JS, Lee KH, et al. Association between painful physical symptoms and clinical outcomes in Korean patients with major depressive disorder: a three-month observational study. Psychiatry Investig. 2009;6(4):255-63. | | |
| --- | --- | --- |
|  | Criteria/Question | Comment |
| **General Quality** | | |
|  | Study Sample Size | The study sample size was 198 enrolled patients, 91/198 patients experienced painful physical symptoms (PPS+), and 107/198 did not (PPS-). |
|  | Respondent selection and recruitment | Patients were enrolled from 7 medical centers including 6 university hospitals throughout Korea. from the psychiatric care setting between June 14, 2006 and February 15, 2007. Patients were followed for a period of 3 months. |
|  | Inclusion/exclusion criteria | Included patients were inpatients or outpatients, aged at least 18 years old, who presented with a new or first episode of MDD, as defined by DSM-IV-TR or ICD-10. Patients had to be prepared to take antidepressant medication. Additionally, patients had to have a CGI-S score ≥430 at study entry, at least 2 months free of depressive symptoms prior to onset of new/first episode and to consent to release of data. Patients were excluded if their current episode of depression had been persistent for more than 6 months, a previously diagnosed other mental health illness or if they were experiencing chronic, treatment-resistant pain. |
|  | Response rates to instrument used | Response rates for EQ-5D are not reported. |
|  | Loss to follow-up | Forty-eight patients were lost to follow-up [28.6% (26/91) PPS+ patients and 20.6% (22/107) PPS- patients]. In the PPS- group, two patients voluntarily discontinued and one patient committed suicide during the study. |
|  | Missing data | Missing data was not reported by the study. |
|  | Any further problems with the study | The researchers identified a number of limitations of the study. Principally, the study was sub-analysis of a larger Asian study, so might not be powered for in-country analysis. Patients without postbaseline visits and measures for all covariates were excluded from the analysis, resulting in a reduced size of the country-specific analysis population. Even though the gender findings were consistent with that of the overall study across all Asian cohorts (68.7% to 78.0% of patients were women who presented PPS with an overall onset of first depressive episode at 41.0 years of age), these elements could be potential confounders. The high unemployment rates observed in this Korean population may be more related to the Korean employment pattern or the higher percentage of unemployed housewives, than the severity of their depression. This could be another confounder. Additionally, the validity of the pain scores used may not be well established and suitably devised for all ages and genders. Additionally, the researchers did not acknowledge that the observational nature of the study was inherently more likely to experience bias in the results. |
|  | Appropriateness of measure | The measures were valid for the population. |
| **Relevance to HTA** | | |
|  | Do the population characteristics in the study match those modeled, and those described in the decision problem of the review? | Yes |
|  | What instrument is used to describe the health states? | EQ-5D |
|  | From which population is the change in HRQoL undertaken? | Directly from the patients. |
|  | From which population is the valuation of changes in the patients’ HRQoL undertaken? | UK general population. |
|  | What technique is used to value the health states? | Not reported by the study. |

Table 16. Utility Weight Quality Assessment of Chen et al. (2010)

| Chen KP, Chiu NY, Shen YC, Hou YM, Yeh CB, Ouyang WC, et al. Association between painful physical symptoms and clinical outcomes in Taiwanese patients with major depressive disorder: A three-month observational study. Asia-Pacific Psychiatry. 2010;2(3):136-45. | | |
| --- | --- | --- |
|  | Criteria/Question | Comment |
| **General Quality** | | |
|  | Study Sample Size | The study sample size was 194 enrolled patients. The patients were categorized in two groups, PPS+, n = 134 (68%) and PPS-, n = 60 (31%). |
|  | Respondent selection and recruitment | Patients were initially enrolled in 6 country observational study in East Asia. This study focuses on the Taiwanese patients from the study, who were recruited from a psychiatric care setting between June 14, 2006 and February 15, 2007. Patients were followed for a period of 3 months. |
|  | Inclusion/exclusion criteria | Included patients were inpatients or outpatients, aged at least 18 years old, who presented with a new or first episode of MDD, as defined by DSM-IV-TR or ICD-10. Patients had to be prepared to take antidepressant medication. Additionally, patients had to have a CGI-S score ≥4 at study entry, at least 2 months free of depressive symptoms prior to onset of new/first episode and to consent to release of data. Patients were excluded if their current episode of depression had been persistent for more than 6 months, a previously diagnosed other mental health illness or if they were experiencing chronic, treatment-resistant pain. |
|  | Response rates to instrument used | Response rates for EQ-5D are not reported. |
|  | Loss to follow-up | In the PPS+ group, 38 patients were lost to follow-up and 11 in the PPS- group. |
|  | Missing data | In addition to those lost to follow-up, in the PPS+ group, 7 patients discontinued of their volition and 7 discontinued due to patient/caregiver decision. In the PPS- group, 1 patient died, 8 patients discontinued of their volition and 4 discontinued due to patient/caregiver decision. |
|  | Any further problems with the study | The researchers identified a number of limitations of the study. Principally, the study was sub-analysis of a larger Asian study, so might not be powered for in-country analysis. The Taiwanese subgroup was relatively small in sample size. The psychiatric setting used in the study may not be representative of all MDD patients. The EQ-5D is based on US and UK populations and has not been validated against Asian populations. Additionally, the researchers did not acknowledge that the observational nature of the study was inherently more likely to experience bias in the results. |
|  | Appropriateness of measure | The measures were valid for the population. |
| **Relevance to HTA** | | |
|  | Do the population characteristics in the study match those modeled, and those described in the decision problem of the review? | Yes |
|  | What instrument is used to describe the health states? | EQ-VAS |
|  | From which population is the change in HRQoL undertaken? | Directly from the patients. |
|  | From which population is the valuation of changes in the patients’ HRQoL undertaken? | Patient population. |
|  | What technique is used to value the health states? | Visual analogue scale. |

Table 17. Utility Weight Quality Assessment of Novick et al. (2015b)

| Novick D, Montgomery W, Moneta V, Peng X, Brugnoli R, Haro JM. Antidepressant medication treatment patterns in Asian patients with major depressive disorder. Patient Prefer Adherence. 2015;9:421-8. | | |
| --- | --- | --- |
|  | Criteria/Question | Comment |
| **General Quality** | | |
|  | Study Sample Size | The study enrolled 909 patients. Included in this sub-analysis were 569 patients who started antidepressant treatment at the baseline visit. |
|  | Respondent selection and recruitment | Patients were initially enrolled in a 6 country observational study in East Asia, who were recruited from a psychiatric care setting between June 14, 2006 and February 15, 2007. Patients were followed for a period of 3 months. Mainland China - n = 300 (33.0%), Hong Kong – n = 89 (9.8%), Malaysia – n = 95 (10.5%), Singapore n = 30 (3.3%), South Korea – n = 197 (21.7%) and Taiwan – n = 198 (21.8%). |
|  | Inclusion/exclusion criteria | Included patients were inpatients or outpatients, aged at least 18 years old, who presented with a new or first episode of MDD, as defined by DSM-IV-TR or ICD-10. Patients had to be prepared to take antidepressant medication. Additionally, patients had to have a CGI-S score ≥4 at study entry, at least 2 months free of depressive symptoms prior to onset of new/first episode and to consent to release of data. Patients were excluded if their current episode of depression had been persistent for more than 6 months, a previously diagnosed other mental health illness or if they were experiencing chronic, treatment-resistant pain. |
|  | Response rates to instrument used | 4/430 (0.9%) did not have EQ-5D response data. |
|  | Loss to follow-up | The loss to follow-up was n=139, 24.4%. |
|  | Missing data | The study did not report on missing data other than those lost to follow-up. |
|  | Any further problems with the study | The researchers identified a number of limitations of the study. The patients were recruited from specialist mental health facilities, so may not be comparable to patients in the primary care setting. Three months follow-up, may not be an appropriate length of time for assessing medication discontinuation. Additionally, The EQ-5D is based on US and UK populations and has not been validated against Asian populations, and the researchers did not acknowledge that the observational nature of the study was inherently more likely to experience bias in the results. |
|  | Appropriateness of measure | The measures were valid for the population. |
| **Relevance to HTA** | | |
|  | Do the population characteristics in the study match those modeled, and those described in the decision problem of the review? | Yes |
|  | What instrument is used to describe the health states? | EQ-5D and EQ-VAS |
|  | From which population is the change in HRQoL undertaken? | Directly from the patients. |
|  | From which population is the valuation of changes in the patients’ HRQoL undertaken? | EQ-5D: UK general population.  EQ-VAS: patient population. |
|  | What technique is used to value the health states? | EQ-5D: time trade-off when using the UK tariff.  EQ-VAS: visual analogue scale. |

Table 18. Utility Weight Quality Assessment of Novick et al. (2015a)

| Novick D, Montgomery W, Moneta MV, Peng X, Brugnoli R, Haro JM. Chinese patients with major depression: do concomitant pain symptoms affect quality of life independently of severity of depression? Int J Psychiatry Clin Pract. 2015;19(3):174-81. | | |
| --- | --- | --- |
|  | Criteria/Question | Comment |
| **General Quality** | | |
|  | Study Sample Size | The main study enrolled 909 patients. Included in this sub-analysis were 300 Chinese patients. |
|  | Respondent selection and recruitment | Patients were initially enrolled in a 6-country observational study in East Asia, who were recruited from a psychiatric care setting between June 14, 2006 and February 15, 2007. Patients were followed for a period of 3 months. This sub-analysis included the patients enrolled from mainland China, n = 300 (33.0% of the total 909 enrolled) |
|  | Inclusion/exclusion criteria | Included patients were inpatients or outpatients, aged at least 18 years old, who presented with a new or first episode of MDD, as defined by DSM-IV-TR or ICD-10. Patients had to be prepared to take antidepressant medication. Additionally, patients had to have a CGI-S score ≥4 at study entry, at least 2 months free of depressive symptoms prior to onset of new/first episode and to consent to release of data. Patients were excluded if their current episode of depression had been persistent for more than 6 months, a previously diagnosed other mental health illness or if they were experiencing chronic, treatment-resistant pain. |
|  | Response rates to instrument used | Response rates for EQ-5D are not reported. |
|  | Loss to follow-up | The loss to follow-up was not reported. |
|  | Missing data | Sixteen patients were missing from the 3-month assessment, but reasons were not reported. |
|  | Any further problems with the study | The researchers identified a number of limitations of the study. Somatic symptoms were collected using a specific questionnaire (SSI), but it is unclear how many of these symptoms would have been reported spontaneously if patients had not been prompted by the questionnaire. The study did not include patients from the primary care setting – only those from psychiatric care settings were included; this means that our sample is not representative of the total MDD population in China and limits the extent to which the findings can be applied to primary care patients with MDD. Comorbidity with anxiety disorders was not assessed but it might be a factor contributing to QoL. Comorbidity with physical medical conditions has not been considered. The analysis was adjusted for whether pain was present or not, but not for severity of pain. Additionally, the EQ-5D is based on US and UK populations and has not been validated against Asian populations, and the researchers did not acknowledge that the observational nature of the study was inherently more likely to experience bias in the results. |
|  | Appropriateness of measure | The measures were valid for the population. |
| **Relevance to HTA** | | |
|  | Do the population characteristics in the study match those modeled, and those described in the decision problem of the review? | Yes |
|  | What instrument is used to describe the health states? | EQ-5D |
|  | From which population is the change in HRQoL undertaken? | Directly from the patients. |
|  | From which population is the valuation of changes in the patients’ HRQoL undertaken? | UK general population. |
|  | What technique is used to value the health states? | Time trade-off when using the UK tariff. |

Table 19. Utility Weight Quality Assessment of Hong et al. (2015)

| Hong J, Novick D, Montgomery W, Moneta MV, Dueñas H, Peng X, et al. Health-related quality of life in patients with depression treated with duloxetine or a selective serotonin reuptake inhibitor in a naturalistic outpatient setting. Patient Prefer Adherence. 2015;9:1481-90. | | |
| --- | --- | --- |
|  | Criteria/Question | Comment |
| **General Quality** | | |
|  | Study Sample Size | A total of 1,647 patients were enrolled, and 1,549 of these patients were classified as “sexually active patients without sexual dysfunction at study entry” and were included in the study. |
|  | Respondent selection and recruitment | This study was a subanalysis of a 6-month multicenter prospective, noninterventional, observational study, primarily designed to examine treatment-emergent sexual dysfunction (TESD) and other treatment outcomes among patients with MDD who were treated with either an SSRI or an SNRI in actual clinical practice. Patients in this study were enrolled from 88 sites between November 15, 2007 and November 28, 2008. Patients were recruited from the following regions: East Asia (People’s Republic of China [n=205; 13.2%], Hong Kong [n=18; 1.2%], Malaysia [n=33; 2.1%], the Philippines [n=113; 7.3%], Taiwan [n=199; 12.8%], Thailand [n=17; 1.1%], and Singapore [n=2; 0.1%]), the Middle East (Saudi Arabia [n=179; 11.6%] and United Arab Emirates [n=135; 8.7%]), Mexico (n=591; 38.2%), and other regions (Israel [n=9; 0.6%] and Austria [n=48; 3.1%]). |
|  | Inclusion/exclusion criteria | Patients (outpatients) were eligible to participate in the study if they met the following inclusion criteria: 1) presenting with an episode of MDD within the normal course of care, with MDD diagnosed according to the ICD-10 or DSM-IV-TR criteria; 2) at least moderately depressed, defined by the CGI-S (with a score of ≥4); 3) initiating or switching to any available SSRI or SNRI antidepressant in any of the participating countries, in accordance with a treating psychiatrist’s discretion; 4) at least 18 years of age; 5) sexually active (with partner or autoerotic activity, including during the 2 weeks prior to study entry) without sexual dysfunction, as defined by Arizona Sexual Experience Scale; 6) not participating in another currently ongoing study; and 7) providing consent to release data. The study excluded the patients who had: 1) a history of treatment-resistant depression (defined as failure to respond to treatment with two different antidepressants from different classes at therapeutic doses for ≥4 weeks); 2) a past or current diagnosis of schizophrenia, schizophreniform or schizoaffective disorder, bipolar disorder, dysthymia, mental retardation, or dementia; or 3) received any antidepressant within 1 week (1 month for fluoxetine) prior to study entry, with the exception of patients receiving an ineffective treatment for whom the immediate switch to an SSRI or SNRI antidepressant was considered to be the best treatment option. Patients who changed or discontinued medication after entry remained in the study, unless lost to follow-up or consent was withdrawn. |
|  | Response rates to instrument used | Response rates for EQ-5D are not reported. |
|  | Loss to follow-up | The loss to follow-up was not reported. |
|  | Missing data | This study included a total of 1,332 patients who 1) initiated either DLX or an SSRI as monotherapy at baseline for the treatment of MDD, and 2) who did not have missing data on the QIDS-SR16 score at baseline with at least one assessable QIDS-SR16 score during follow-up (n=556 in the DLX group and n=776 in the SSRI group). This study analyzed the patient observations up to the point where their initial medications were discontinued. Of the 1,332 patients, 78.7% (n=1,048) were available at 24 weeks (n=443 [79.7%] in the DLX group and n=605 [78.0%] in the SSRI group). |
|  | Any further problems with the study | The researchers acknowledged that as the study was observational, it did not imply causal relationships, as it possible that not all potential sources of bias were accounted for by the study analysis. The researchers also acknowledged that the objective of assessing TESD, and the inclusion of only sexually active MDD patients with no sexual dysfunction meant that the results of the study may not be generalizable to the wider MDD population. Additionally, as EQ-5D tariffs were not available for all countries included in the study, for the analysis the UK tariff was applied. |
|  | Appropriateness of measure | The measures were valid for the population. |
| **Relevance to HTA** | | |
|  | Do the population characteristics in the study match those modeled, and those described in the decision problem of the review? | Yes |
|  | What instrument is used to describe the health states? | EQ-5D |
|  | From which population is the change in HRQoL undertaken? | Directly from the patients. |
|  | From which population is the valuation of changes in the patients’ HRQoL undertaken? | UK general population. |
|  | What technique is used to value the health states? | Time trade-off when using the UK tariff. |

Table 20. Utility Weight Quality Assessment of Kim et al. (2015)

| Kim JM, Chalem Y, di Nicola S, Hong JP, Won SH, Milea D. A cross-sectional study of functional disabilities and perceived cognitive dysfunction in patients with major depressive disorder in South Korea: the PERFORM-K study. Psychiatry Res. 2016;239:353-61. | | |
| --- | --- | --- |
|  | Criteria/Question | Comment |
| **General Quality** | | |
|  | Study Sample Size | The study enrolled 343 patients, of which 312 were included in the analysis population. Fourteen patients were excluded due to at least one inclusion/exclusion criterion was not respected, and 27 patients were excluded due to at least one exclusion criterion being met. |
|  | Respondent selection and recruitment | Patients were recruited from 29 psychiatric departments in university or general hospitals throughout South Korea from October 2013 to January 2014. |
|  | Inclusion/exclusion criteria | Eligible patients were outpatients between 19 and 65 years old with a diagnosis of MDD according to the DSM-IV, confirmed by the Mini-International Neuropsychiatric Interview (Sheehan etal.,1998), and started on antidepressant monotherapy either as first-line therapy or as first treatment switch from previous antidepressant monotherapy. Patients were excluded if they: had a diagnosis of bipolar disorder, schizophrenia or other psychotic disorder, substance dependence, dementia or other neurodegenerative diseases that affect cognitive functioning, or a mood disorder due to general medical conditions or substances; were acutely suicidal; were pregnant, breast feeding or 6 months post-partum; were unable to read or understand the information sheet, informed consent form or patient-reported questionnaires ;or were concurrently participating in another clinical trial. Based on the above inclusion and exclusion criteria, patients attending psychiatric departments of the study hospitals were recruited consecutively if they agreed to participate. |
|  | Response rates to instrument used | Response rates to EQ-5D were not presented, only baseline data. |
|  | Loss to follow-up | The loss to follow-up was not reported. |
|  | Missing data | Imputation of missing data was not used in any of the analyses (i.e. all data were observed cases). Two patients were missing from the health state score. |
|  | Any further problems with the study | The researchers did not acknowledge that the observational nature of the study was inherently more likely to experience bias in the results. However, the researchers acknowledged that their instrument used for assessing subjective cognitive decline rather than objective cognitive decline. Additionally, the study only recruited patients initiating a new antidepressant monotherapy treatment, so the results may not be generalizable to all MDD patients in South Korea. |
|  | Appropriateness of measure | The measures were valid for the population. |
| **Relevance to HTA** | | |
|  | Do the population characteristics in the study match those modeled, and those described in the decision problem of the review? | Yes |
|  | What instrument is used to describe the health states? | EQ-5D |
|  | From which population is the change in HRQoL undertaken? | Directly from the patients. |
|  | From which population is the valuation of changes in the patients’ HRQoL undertaken? | Not reported by the study. |
|  | What technique is used to value the health states? | Not reported by the study. |

Table 21. Utility Weight Quality Assessment of Fernandez et al. (2005)

| Fernandez JL, Montgomery S, Francois C. Evaluation of the cost effectiveness of escitalopram versus venlafaxine XR in major depressive disorder. Pharmacoeconomics. 2005;23(2):155-67. | | |
| --- | --- | --- |
|  | Criteria/Question | Comment |
| **General Quality** | | |
|  | Study Sample Size | The study sample size was 293 outpatients with moderate to severe MDD. |
|  | Respondent selection and recruitment | Patients were recruited to a randomized, double-blind, flexible-dose, multinational clinical trial. The paper did not explicitly say how the patients were recruited or from which countries, but the paper mentions the UK, Germany, France, Spain, Denmark and Finland. |
|  | Inclusion/exclusion criteria | Patients aged 18–85 years fulfilling DSM-IV criteria for moderate to severe MDD, without suicidal tendencies. The patient’s MADRS total score was required to be ≥18 at screening 1 week before the start of treatment and at start of treatment. Patients were excluded if they met any of the following criteria: history of mania or any bipolar disorder, schizophrenia or any psychotic disorder, or current evidence of OCD, eating disorders, mental retardation, any pervasive serotonin development disorder or cognitive disorder. Patients taking medications thought likely to interfere with the study were excluded. |
|  | Response rates to instrument used | The percentage of patients reporting at least some problems for EQ-5D dimensions were reported at baseline and week 8. But raw scores were not reported for week 8. |
|  | Loss to follow-up | The loss to follow-up was not reported. |
|  | Missing data | Given the very low rate of attrition in the sample during the trial, patients with missing data were unlikely to represent serious bias to the results of the present analysis. As a result, no attempt was made to impute missing data. |
|  | Any further problems with the study | The researchers acknowledged that the sample size of the study was calculated based on the MADRS total score in the clinical trial. Larger sample sizes are required to increase the power of performed tests. The utilities for MDD were not the primary objective of the study, so information regarding the patient population was sparse. |
|  | Appropriateness of measure | The measures were valid for the population. |
| **Relevance to HTA** | | |
|  | Do the population characteristics in the study match those modeled, and those described in the decision problem of the review? | Yes |
|  | What instrument is used to describe the health states? | EQ-5D |
|  | From which population is the change in HRQoL undertaken? | Directly from the patients. |
|  | From which population is the valuation of changes in the patients’ HRQoL undertaken? | UK general population |
|  | What technique is used to value the health states? | Time trade-off when using the UK tariff. |
